# Synthesis, Characterization, and Cytotoxicity of Dicyclo­alkyl­amine­pyrophosphato­platinum​(II) Complexes

**DOI:** 10.1021/acsomega.5c07828

**Published:** 2026-02-10

**Authors:** Dianne M. Wagner, Dieu Huyen My Nguyen, Emily McHenry, Lanise A. Brown, Taylor Lindholm, Sarita S. Yadav, Glenn P. A. Yap, Michael J. Toneff, Robert J. Mishur

**Affiliations:** † Department of Chemistry, One University Pl, 2392 Widener University, Chester, Pennsylvania 19013, United States; ‡ Department of Biology, One University Pl, Widener University, Chester, Pennsylvania 19013, United States; § Department of Chemistry and Biochemistry, 5972 University of Delaware, Newark, Delaware 19716, United States

## Abstract

Platinum complexes have now been used in chemotherapy regimens for almost half a century to treat a variety of cancers. The most clinically significant of these compounds to date is cisplatin, *cis*-di­ammine­dichloro­platinum­(II), whose clinical application has significantly reduced the mortality rate of several cancers. Despite this development, there is still a push to find new compounds that have improved efficacy, that can be administered at lower doses, and that produce less severe side-effects compared to current options. One class of molecules that may fill that role is phosphaplatins, an underexplored class of platinum­(II) complexes that contain a bidentate pyrophosphate ligand. These compounds are anionic at physiological pH and display reduced DNA-binding compared to cisplatin. This study expands on the list of known phosphaplatins by introducing two new compounds, di­cyclo­butyl­amine­dihydrogen­pyro­phosphato­platinum­(II) and dicyclo­pentylamine­dihydrogen­pyrophosphato­platinum­(II). Here we report complete synthetic details, as well as cell viability data in response to these compounds using two cancer cell lines, a human lung adenocarcinoma and a triple-negative human breast cancer. While these compounds inhibit cell viability less than the leading phosphaplatin drug candidate, *trans*-(1*R*,2*R*)-diamino­cyclo­hexane­dihydrogen­pyro­phosphato­platinum­(II), this work represents an important step in elucidating structure–activity relationships for this class of molecules.

## Graphical abstract

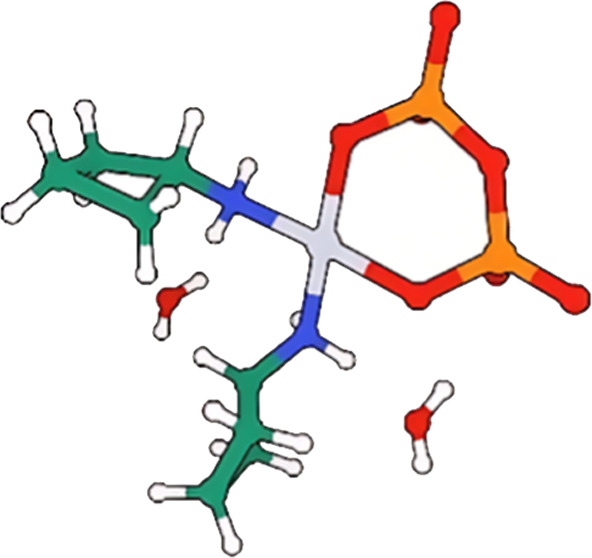

## Introduction

Cisplatin, *cis*-diammine­dichloro­platinum­(II), is a potent antitumor agent that has been used in chemotherapy-based treatments for testicular, ovarian, small-cell lung, head and neck, breast, uterus, cervix, and bladder cancers.^1^ Notably, its use has significantly increased the cure rate for testicular cancer from less than 10 to over 90%.^2^ However, there are severe drawbacks to treatment with cisplatin including side effects, such as nausea, vomiting, hair loss, hearing loss, and kidney toxicity. Additionally, certain tumors can display intrinsic or acquired resistance to cisplatin following prolonged treatment.^3^ Therefore, it is imperative to find alternatives that are less toxic, that are effective at lower doses, and that do not engender cross-resistance with existing drugs. This has led to widespread screening of tens of thousands of platinum compounds for anticancer activity over the past five decades. Despite this effort, only three such compounds have been approved for clinical use in the U.S (cisplatin, carboplatin, and oxaliplatin; Figure 1), with three additional compounds having been approved for regional use in Asia (nedaplatin, lobaplatin, and heptaplatin).^4^ Each of the approved compounds distort the structure of DNA by binding to purine bases, causing inhibition of cellular processes such as DNA replication, transcription and translation resulting in cell cycle arrest and apoptosis.^5,6^

**Figure 1:**
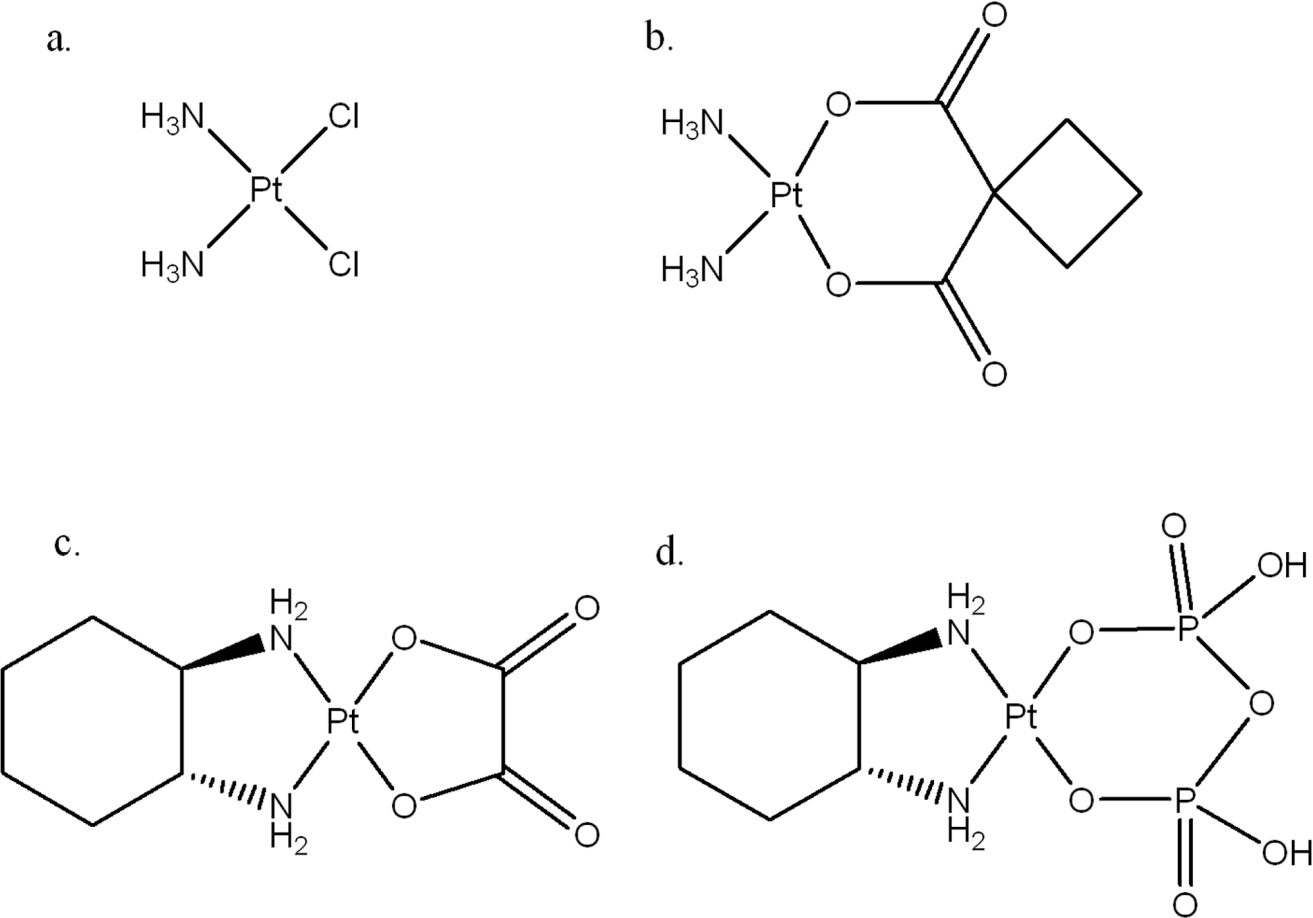
Structures of FDA-approved platinum drugs: (a) cisplatin, (b) carboplatin, and (c) oxaliplatin, and (d) leading phosphaplatin candidate RRD2 (PT112).

Phosphaplatins, which are defined as having a bidentate pyrophosphate group complexed to a platinum center with two N donor ligands (or a bidentate N,N donor ligand) are an emerging class of platinum drugs with potent antitumor activity.^7–10^ The leading phosphaplatin drug candidate, *trans*-(1*R*,2*R*)​diamino­cyclohexane­dihydrogen­pyro­phosphato­platinum​(II) (RRD2, also known as PT112; Figure 1d), is currently undergoing phase I/II clinical trials in patients with advanced solid tumors (NCT02266745). However, this class of molecules still remains relatively unexplored. Further, existing reports suggest that these molecules may have mechanisms of action which differ from traditional platinum-based drugs, including a reduced tendency to form covalent adducts with DNA, likely due to electrostatic interactions between the anionic platinum compounds and the DNA phosphate backbone.^7,11,12^ Thus, it remains unclear whether these compounds follow established structure-based activity relationships for platinum compounds.

In this study, we set out to synthesize a series of compounds of the type dialkyl­amine­pyro­phosphato­platinum­(II), because structure/activity relationships for di­alkylamine­dichloro­platinum­(II) compounds are already established.^13^ Here, we report on the synthesis, characterization, and physical properties of two new monomeric platinum-pyro­phosphato compounds: *cis*-dicyclo­butyl­amine­dihydrogen­pyrophosphato­platinum­(II) (cBuAm-2) and *cis*-di­cyclo­pentyl­amine­dihydrogen­pyro­phosphato­platinum­(II) (cPnAm-2). We also report the crystal structure of a dinuclear platinum­(II)-pyro­phosphato complex, which has the potential to serve as a prodrug for the monomeric complex.^8^

## Experimental Section

### Reagents

Sodium pyrophosphate decahydrate (≥99%) was purchased from Sigma-Aldrich and recrystallized from boiling deionized water. Potassium tetra­chloroplatinate (≥99.9%), cyclo­butyl­amine (98%), adenosine 5′-mono­phosphate (≥97%) (AMP), and deuterium oxide (99.9% D) were purchased from Sigma-Aldrich (St. Louis, MO). Silver nitrate (99.9+%), cyclopentylamine (99%), *trans*-1,2-diamino­cyclo­hexane (99%), l-cysteine (98+%), l-glutathione (98+%), 2′-deoxyguanosine-5′-monophosphate (98%) (dGMP), lithium perchlorate (ACS grade, ≥95%), potassium iodide (99%), and inorganic pyrophosphatase (Thermo EF0221) were purchased from Thermo Fisher Scientific (Waltham, MA). All other reagents were of the highest purity available and used without further purification.

### Elemental Analysis

C, H, and N elemental combustion analyses were performed by Galbraith Laboratories, Knoxville, TN.

### pH Measurements

pH measurements were performed using a Milwaukee Pro MW102 pH meter with a Sigma-Aldrich micro pH Ag/AgCl combination electrode (Sigma Z113441). A two-point calibration was performed using standard buffer solutions prior to all pH measurements.

### Nuclear Magnetic Resonance Spectroscopy

NMR measurements were carried out on a Bruker Avance 400 MHz spectrometer. ^31^P NMR spectra were recorded with respect to an external standard of 85% phosphoric acid. ^1^H and ^13^C NMR spectral shifts were recorded with respect to tetra­methyl­silane. Deuterium oxide was used to provide a lock signal. For acidity constant measurements, an internal capillary of D_2_O was used to avoid having to apply corrections to pH measurements. ^31^P NMR spectra were acquired with typical parameters: 6.5 kHz sweep width and a 90° pulse of 2.2 μS, with a 2.0 s delay between pulses. Free inductive decays (FIDs) consisting of 32 000 data points were collected, and a 3.0 Hz line broadening factor was applied following Fourier transformation. For 10 mM solutions of platinum complexes, 64 scans were typically sufficient to produce spectra with a signal-to-noise ratio (S/N) greater than 10. Acquisition parameters for ^13^C NMR were 16 000 scans with a 24 kHz sweep width and a 2.0 s delay between pulses. ^1^H NMR were acquired with between 32 and 128 scans with a 6.4 kHz sweep width and a 0.3 s delay between pulses. Some ^1^H NMR spectra were acquired using the zgcppr water suppression pulse sequence with a 28 dB presaturation pulse over the resonance of the water peak and a 2 s delay between 90° pulses.

### Mass Spectrometry

Electron spray ionization mass spectrometry (ESI–MS) was performed with a Finnigan LTQ LC/MS/MS (Thermo-Fisher Scientific, Waltham, MA, U.S.A.). Solutions were prepared for analysis by dissolving compounds in a 90:9:1 solution of deionized water, ethanol, and acetic acid, respectively. Samples were loaded by a syringe pump directly into the spectrometer. The nozzle was set to a high voltage, typically within several kilovolts of 4.5 kV. All spectra were acquired in positive ion mode.

### X-ray Diffraction Measurements

Crystals of μ-pyro­phosphato­tetrakis­(cyclo­butyl­amine)­diplatinum­(II) dihydrate were grown by allowing a dilute acidified aqueous solution to sit undisturbed for several weeks. Crystals of sodium dicyclo­butyl­amine­pyro­phosphato­platinate­(II) trihydrate were grown by layering *N*,*N*-dimethyl­formamide onto an alkaline aqueous solution. Candidate data crystals were selected, sectioned, mounted using viscous oil onto plastic mesh and cooled to the data collection temperature. Data were collected on a D8 Venture Photon diffractometer with Cu–Kα radiation (λ = 1.54178 Å) focused with Goebel mirrors. Unit cell parameters were obtained from fast scan data frames, 1°/s ω, of an Ewald hemisphere. The unit-cell dimensions, equivalent reflections and systematic absences in the diffraction data were consistent with *Pnma* and *Pna*2_1_ for μ-pyro­phosphato­tetrakis­(cyclo­butyl­amine)­diplatinum­(II) dihydrate, and the centrosymmetric option yielded chemically reasonable and computationally stable results of refinement. For sodium di­cyclo­butyl­amine­pyro­phosphato­platinate­(II) trihydrate, no symmetry higher than triclinic was observed, and the centrosymmetric option, *P*-1, yielded chemically reasonable and computationally stable results of refinement. The data were treated with multiscan absorption corrections.^14^ The structures were solved in Olex2 ^15^ using intrinsic phasing methods^16^ and refined with full-matrix, least-squares procedures on *F* ^2^.^17^

### Synthesis of Di­chloro­dicyclo­alkyl­amine­platinum­(II) Complexes

*Cis*-dichloro­dicyclo­butyl­amine­platinum­(II) and *cis*-dichloro­dicyclo­pentyl­amine­platinum­(II), were synthesized using a modified procedure initially reported by Braddock et al.^13^ Potassium tetra­chloro­platinate (∼1.5 g, 3.6 mmol) was dissolved in 15 mL of deionized water, and the solution was gravity filtered to remove any insoluble impurities. Following filtration, 5 mL of ethanol was added, resulting in a pink precipitate that redissolved on stirring. Once the solution was clear, 2 mol equiv of either cyclobutylamine or cyclopentylamine were added via micropipette. Reaction mixtures were allowed to stir at room temperature overnight, during which time a pale-yellow precipitate formed (it should be noted that the longer the reaction proceeds, the less pure the isolated product is, as evidenced by a “dingy” appearance; it is recommended to collect an initial crop of precipitate after a few hours of reaction time if a higher purity is needed; in particular, using a less pure crop of dichlorodicyclopentylamineplatinum­(II) in the reaction with pyrophosphate results in a diminished yield and difficulty isolating the product). Products were isolated by vacuum filtration on a glass frit and washed with approximately 3 mL each of concentrated HCl, deionized water, methanol, and diethyl ether, in sequence. Both racemic *trans*-1,2-diamino­cyclo­hexane­dichloro­platinum­(II) and the stereochemically pure *trans*-(1*R*,2*R*)-di­amino­cyclo­hexane­dichloro­platinum­(II) were prepared by making appropriate modifications to the synthesis of Dhara^18^ as previously reported.^19^ Yield *cis*-di­chloro­dicyclo­butyl­amine­platinum­(II) 38%. Yield *cis*-di­chloro­dicyclo­pentyl­amine­platinum­(II) 43%.

### Synthesis of Dicylco­alkyl­amine­pyro­phosphato­platinum­(II) Complexes

Di­cyclo­alkyl­aminedi­hydrogen­pyro­phosphato­platinum­(II) complexes, racemic *trans*-1,2-diamino­cyclo­hexane­dihydrogen­pyro­phosphato­platinum­(II) (dach-2), and *trans*-(1*R*,2*R*)-diamino­cyclohexane­di­hydrogen­pyro­phosphato­platinum­(II) (RRD2) were synthesized by making modifications to the procedure of Mishur and Bose.^19^ Tetrasodium pyrophosphate decahydrate (0.4 g) was dissolved in 200 mL of a 4% v/v ethanol/water solution, and the pH was lowered to 8.0 by addition of approximately 20 drops of 1 M HNO_3_. *Cis*-dichlorodicycloalkylamine complexes were added (0.1 g), and the reaction mixtures were incubated for 15 h at 60 °C or until all solids were dissolved, up to 72 h.

Following the incubation period, solutions were concentrated to approximately 5 mL by rotary evaporation and filtered as necessary. Filtrates were chilled on ice for 10 min, and the pH was then adjusted to pH 1–2 by addition of 1 M HNO_3_. Following the initial appearance of a white precipitate, reaction mixtures were kept on ice for an additional 10 min to complete precipitation of the desired products. Products were then isolated by vacuum filtration on a glass frit and washed with 1–2 mL portions each of ice-cold water, ethanol, and diethyl ether. Yield cBuAm-2 33%; ESI *m*/*z* = 514 ([M + H]^+^), 536 ([M + Na]^+^); ^31^P NMR 1.58 ppm. Elemental analysis: expected C 18.79%, H 3.55%, N 5.48%; found, C 18.32%, H 3.82%, N 5.45%. Yield cPnAm-2 23%; ESI *m*/*z* = 542 ([M + H]^+^), 564 ([M + Na]^+^); ^31^P NMR 1.37 ppm. Elemental analysis: expected C 22.19%, H 4.11%, N 5.19%; found, C 21.23%, H 4.15%, N 5.06%. Yield dach-2 39%.

### Acidity Constant Measurements

Dicyclo­amine­dihydrogen­pyro­phosphato­platinum­(II) compounds were dissolved in 1 M NaOH to produce 10 mM aqueous solutions with pH ∼13 and analyzed by ^31^P NMR spectroscopy. An internal capillary of D_2_O was used to provide the deuterium lock, to avoid having to make pH corrections. The pH of solutions was lowered by incremental addition of 0.1 M HNO_3_, and ^31^P NMR spectra were acquired every ∼1 pH unit until a pH of 1 was reached. Acidity constants were determined by fitting this data to the equation of a diprotic acid (eq 1)^20^ using SigmaPlot 11.0.^21^

1 $$\delta =\frac{\delta_{1}\left[H_{3}O^{+}\right]+\delta_{2}Ka\left[H_{3}O^{+}\right]+\delta_{3}{Ka}_{1}{Ka}_{2}}{{\left[H_{3}O^{+}\right]}^{2}+Ka\left[H_{3}O^{+}\right]+{Ka}_{1}{Ka}_{2}}$$

In eq 1, *K*a_1_ and *K*a_2_ represent the first and second acidity constants.^20^ The observed ^31^P chemical shift (δ) vs hydronium ion concentration was fit to this equation, and the chemical shifts of the diprotic, monoprotic, and unprotonated forms of the platinum complex are represented by δ_1_, δ_2_, and δ_3_, respectively.

### Aqueous Stability Study

A study was performed to determine how long di­cyclo­alkyl­amine­pyro­phosphato­platinate​(II) ions are stable in aqueous solution. Aqueous solutions of 10 mM cBuAm-2 were prepared in 1 M sodium hydroxide and a few drops of D_2_O. The pH of each solution was then adjusted to either 7.0, 5.5, or 4.3 using 0.1 M HNO_3_. Solutions were allowed to stand at room temperature, and a ^31^P NMR spectrum was recorded every 24 h for 1 week.

### Reactions with Pyro­phosphatase

The reaction of cBuAm-2 with pyro­phosphatase was examined to determine whether the presence of the enzyme can cleave coordinated pyrophosphate (note–we use the same abbreviations for phosphaplatins whether they are in acid or base form; these compounds are isolated as diprotic acids and are dianions in the pH range in which our experiments were performed). Either tetrasodium pyrophosphate or cBuAm-2 (4 mM) was dissolved in 100 mM TrisHCl buffer (pH 7.2) containing 2 mM magnesium chloride, and a few drops of D_2_O were added. After adding 50 μL of pyrophosphatase (0.1 U/μL) into each solution, the reactions were monitored by ^31^P NMR spectroscopy (1 unit of pyro­phosphatase represents the amount needed to release 1 μmol of orthophosphate per minute at pH 9 and 25 °C).

### Nucleotide Binding

The reactions of phosphaplatins with purine bases deoxyguanosine monophosphate (dGMP) and adenosine mono­phosphate (AMP) were examined. Reaction mixtures were prepared containing 2 mM platinum (as either cBuAm-2, cPnAm-2, or dach-2) and 100 mM lithium perchlorate to maintain constant ionic strength, with final volumes of 5 mL. The pH was adjusted to 7.5 using 1 M sodium hydroxide, and 0.025 mmol of solid nucleotide (dGMP or AMP) and a few drops of D_2_O were added. Reactions were monitored by water-suppressed ^1^H NMR for 48 h. A ^31^P NMR spectrum was obtained at the end of 48 h. Final pH was not recorded.

A binding competition study was performed by combining platinum complexes with a mixture containing equal amounts of dGMP and AMP. Reaction mixtures contained 5 mM of platinum complex (as either cBuAm-2, racemic dach-2, or cisplatin), 100 mM lithium perchlorate, and 10 mM each of dGMP and AMP in D_2_O. The pH* (uncorrected pH measurement) was adjusted to 7.4 using 3 M sodium hydroxide at the beginning of the experiment. Reaction progress was monitored by ^1^H NMR every 24 h for up to 1 week. Between active NMR scans, the samples were kept in a 37 °C water bath.

### Reactions with Thiols

The kinetics of the reactions of dicyclo­alkyl­amine­pyrophophato­platinate­(II) ions with cysteine and glutathione were examined using pseudo-first order reaction conditions. Reaction mixtures contained 3 mM Pt (as either cBuAm-2 or cPnAm-2), 60 mM of either cysteine or glutathione, 30% deuterium oxide, 0.1 M lithium perchlorate to maintain ionic strength, and 0.1 M Bis-Tris as a buffer. The pH of reactions was adjusted to 7.5 using 0.1 M sodium hydroxide and reaction progress was monitored by ^31^P NMR for 500 min at 10 min intervals. The NMR probe heater was utilized to maintain temperatures at a constant at 25 °C for the duration of the experiments. Pseudo-first order rate constants were obtained by plotting the natural log of the integral of the decaying ^31^P signal from the platinum complex (as a percentage of overall peak area) versus time and determining the slope of the trendline.

### Cell Culture

MDA-MB-231 (triple-negative breast cancer) and A549 (nonsmall cell lung adenocarcinoma) cells were used for cytotoxicity studies. MDA-MB-231 cells were obtained from the Tissue and Cell Culture Core Laboratory at Baylor College of Medicine (Houston, TX), originally sourced from the American Type Culture Collection (ATCC; Manassas, VA). A549 cells were purchased directly from ATCC. MDA-MB-231 cells were cultured in high-glucose Dulbecco’s modified Eagle’s medium (DMEM), and A549 cells were cultured in Ham’s F-12 K (Kaighn’s) medium. Both media were supplemented with 10% fetal bovine serum (FBS) and 1% antibiotic/antimycotic. Cells were passaged to maintain exponential growth and incubated at 37 °C in a humidified incubator with 5% CO_2_ and ambient O_2_.

### Viability Assay

Cytotoxicity was assessed using the MTT assay (Promega, TB112) according to the manufacturer’s specifications. Cells were seeded in 96-well plates in 100 μL of growth medium and allowed to adhere overnight. MDA-MB-231 cells were seeded at 5000 cells/well for cisplatin treatment and 3000 cells/well for phosphaplatins, while A549 cells were seeded at 3000 cells/well for cisplatin and 2000 cells/well for phosphaplatins. Cells were treated with either cisplatin, RRD2, cBuAm-2, or cPnAm-2 at concentrations ranging from 3.125–100 μM for cisplatin (48 h), 0.274–200 μM for RRD2 (72 h), and 6.25–200 μM for cBuAm-2 and cPnAm-2 (72 h). Stock solutions were prepared in phosphate-buffered saline (PBS) and diluted in culture medium. Each condition was tested in triplicate biological replicates, with six technical replicates per biological replicate under each condition.

MTT assay absorbance was measured using a Synergy H4 microplate reader (BioTek Instruments, Inc., Winooski, VT, U.S.A.). Vehicle-treated controls were normalized to 100% cell viability and mean % viability at each compound concentration for each biological replicate was determined. The mean % viability was used to calculated the relative IC_50_ values for each compound. Relative IC_50_ values were determined by nonlinear regression using GraphPad Prism MacOS version 10.5.0, GraphPad Software, Boston, MA, U.S.A., www.graphpad.com.^22^

### Safety Considerations

Care should be taken when handling platinum compounds, including newly discovered compounds, as many of these species can covalently bind to DNA, inducing mutations and carcinogenesis. Therefore, all platinum compounds should be treated as potential carcinogens.

## Results and Discussion

### Synthesis and Characterization of Di­cyclo­alkylamine­di­hydrogen­pyro­phosphato­platinum­(II) Compounds

Di­cyclo­alkyl­amine­dihydrogen­pyro­phosphato­platinum­(II) complexes were prepared from *cis-*dichlorodicycloalkylplatinum­(II) compounds using a modified version of the method developed by Mishur and Bose.^19^ Due to poor aqueous solubility of *cis*-dichloro­dicyclo­alkyl­platinum­(II) species, owing to the highly lipophilic nature of the cycloalkyl groups, up to 4% ethanol was added as a wetting agent.^23^ Products were characterized by ^1^H, ^13^C, and ^31^P NMR spectroscopy, and, by ESI-MS. As expected, due to the symmetry of the bidentate pyrophosphate group, both cBuAm-2 and cPnAm-2 displayed a single peak in the ^31^P NMR spectrum. These peaks appear downfield from the peak for free pyrophosphate because of the strong deshielding effect of the Pt­(II) nucleus. No satellites were observed in the ^31^P NMR spectra from coupling to ^195^Pt. Molecular weights were confirmed by ESI-MS, and both [M + H]^+^ and [M + Na]^+^ peaks were observed for cBuAm-2 and cPnAm-2. Representative spectra are shown in Supplemental Figures S1–S8. Purity was assessed through elemental analysis by an outside laboratory. For cPnAm-2, we found good agreement between expected and observed values for CHN analysis. However, for cBuAm-2, the measured value for %C was 0.47% lower than calculated, while %N was within 0.3% of the calculated value and %H was within 0.05% of the calculated value. Since these compounds are selectively precipitated from solution, it is unlikely that there would be significant amounts of impurities. Indeed, elemental analysis of other phosphaplatins synthesized by similar methods results in good agreement between calculated and expected values.^8^ It is unclear why our %C deviates from the expected value; however, due to the consistency of the calculated and found values for %H and %N, along with the fact that the molar mass determined by ESI-MS agrees with the expected mass, we suspect that this is perhaps an error arising from the methods employed by the analytical laboratory.

Crystals of cBuAm-2 were grown by carefully layering *N*,*N*-dimethyl­formamide on an aqueous basic solution inside an NMR tube. Figure 2 shows the X-ray crystal structure for cBuAm-2. The compound is an ionic chain polymer propagated in the crystal by inversion with interactions between sodium ions, phosphate ions, and water molecules. Interchain interactions orthogonal to the chain direction are H-bonding interactions in one direction and van der Waals attractions between cyclobutyl groups of the Pt­(NH_2_-cyclobutyl)_2_ moieties pendent to the chain in the other direction (Figure 2, bottom panel). The cyclobutyl groups were refined with three-dimensional anisotropic displacement rigid-group restraints. Water molecules were treated to idealized geometry based on initial locations of the H atoms from the electron density difference map with *U*_iso_ equal to 1.5 *U*_eq_ of the attached oxygen atom.

**Figure 2:**
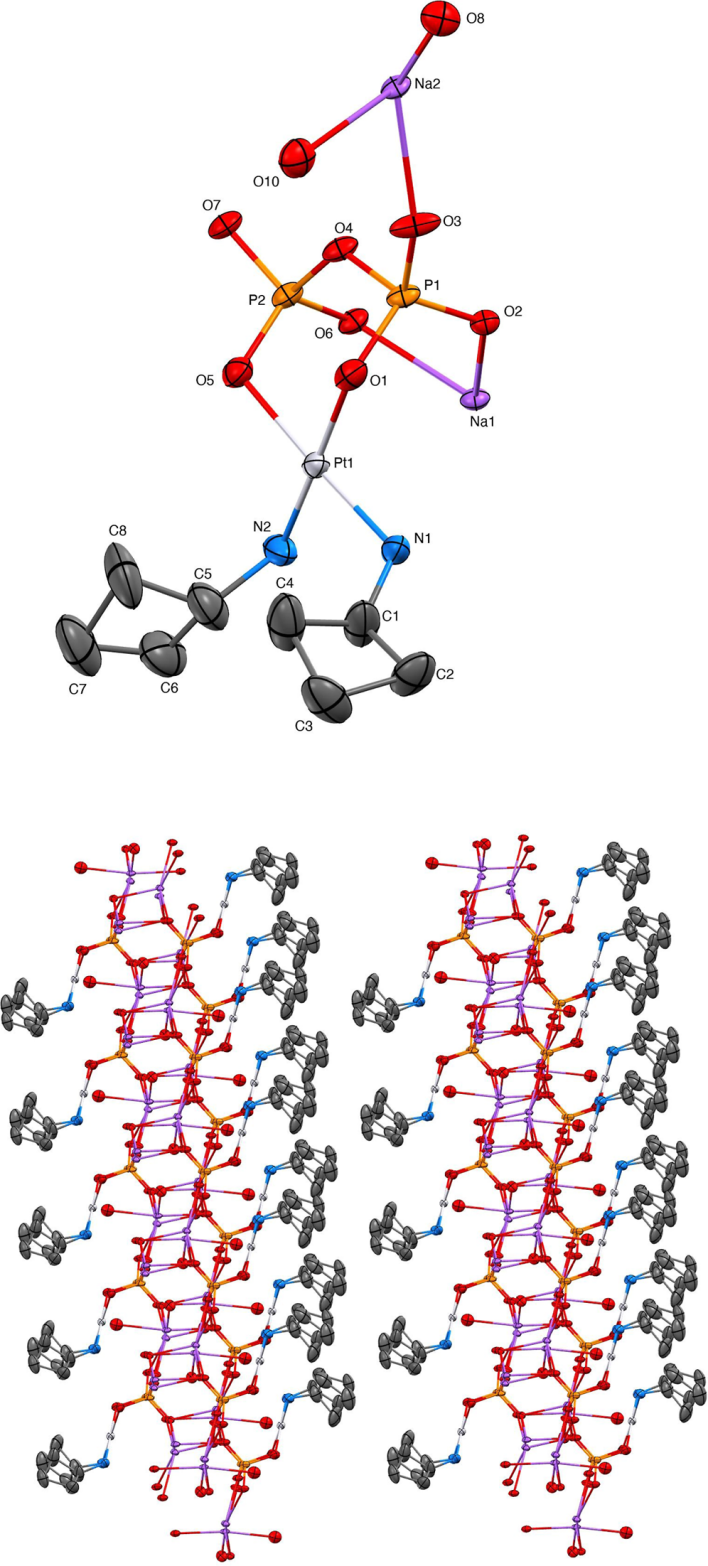
(Top) X-ray crystal structure of cBuAm-2. (Bottom) Extended crystal structure showing H-bonding and van der Waals interactions between cBuAm-2 moieties.

A sodium ion, oxide and water moiety, and a water molecule were located disordered in two positions with a refined site occupancy ration of 60/40 and 70/30, respectively. Disordered contributions were constrained with equal atomic displacement between corresponding atom pairs. Non-hydrogen atoms were refined with anisotropic displacement parameters. Hydrogen atoms, other than the water H atoms, were treated as idealized contributions with geometrically calculated positions and with *U*_iso_ equal to 1.2 *U*_eq_ of the attached carbon atom. Atomic scattering factors are contained in the SHELXTL program library.^17^ The crystal structure has been deposited at the Cambridge Structural Database under CCDC 2465450, DOI: 10.5517/ccdc.csd.cc2nrhnl. Crystal data and refinement details are summarized in Table 1.

**Table 1:** Crystal Data and Structure Refinement Details

compound		sodium dicyclo­butylamine-pyro­phosphato-platinate(II) trihydrate	(μ-pyro­phosphato)-tetrakis­(cyclo­butyl­amine)-diplatinum(II) dihydrate
sum formula		C_8_H_24_N_2_Na_2_O_10_P_2_Pt	C_16_H_40_N_4_O_9_P_2_Pt_2_
moiety formula		C_8_H_18_N_2_Na_2_O_8_P_2_Pt, 3(H_2_O)	C_16_H_36_N_4_O_7_P_2_Pt_2_, 2(H_2_O)
formula weight, g/mol		611.30	884.64
temperature, K		100.00	100.00
crystal system		triclinic	orthorhombic
space group		*P*-1	*Pnma*
cell dimensions			
	*a*, Å	5.6758(7)	15.197(2)
	*b*, Å	9.3516(12)	24.101(3)
	*c*, Å	18.010(2)	7.5399(10)
	α, °	90	99.974(4)
	β, °	90	95.922(4)
	γ, °	90	102.098(4)
	volume, Å^3^	910.9(2)	2761.6(7)
*Z*		2	4
ρ_calc_, g/cm^3^		2.229	2.128
μ, mm^–1^		17.030	20.199
*F*(000)		592.0	1688
reflections collected		19680	14540
independent reflections		3437	2688
data/restraints/parameters		3437/135/249	2688/43/167
goodness-of-fit		1.047	1.040
*R* [*I* ≥ 2σ(*I*)] *R*_1_/*wR*_2_		*R*_1_ = 0.0361, w*R*_2_ = 0.0919	0.0890/0.2620
*R* indexes [all data] *R*_1_/*wR*_2_		*R*_1_ = 0.0380, w*R*_2_ = 0.0932	0.1040/0.2740
CCDC		2465450	2302238

We also synthesized dicyclo­propyl­amine­pyrophosphato­platinate­(II) (cPrAm-2) *in situ* (^31^P NMR 1.97 ppm at pH 8, Figure S9); however, attempts at isolating the acid from solution were unsuccessful. This reaction is difficult to reproduce. The compound *cis*-dichloro­dicyclo­propyl­amine­platinum­(II) is not formed as a pure solid by addition of KCl to *cis*-diaqua­dicyclo­propyl­amine­platinum­(II) at 40 °C, as evidenced by the dark color of the product. A pale-yellow product is obtained if the reaction is not heated. Impure *cis*-dichloro­dicyclo­propyl­amine­platinum­(II) can be recrystallized from boiling 1 M HCl to obtain a light-yellow solid, but purification does not seem to affect the outcome of the next reaction. Following the reaction with tetrasodium pyrophosphate, a peak at 3.81 ppm (at pH 8) in the ^31^P NMR spectrum is frequently observed as the only product. This is attributed to orthophosphate, PO_4_^3–^, resulting from Pt­(II) catalyzed hydrolysis of pyrophosphate.^24^ It is unclear why this does not occur in reactions involving cyclobutylamine or cyclopentylamine. It is noted that the ^31^P chemical shift trend (recorded at pH 8:1.97 ppm, cPrAm-2; 1.58 ppm, cBuAm-2; 1.37 ppm, cPnAm-2) mirrors what is expected based on the chemical structures. When increasing the number of carbons in the cycloalkylamine ligands, a greater inductive effect is observed, with increased electron density around the phosphorus atoms resulting in an upfield chemical shift. We were unsuccessful at preparing dicyclo­hexyl­amine­dihydrogen­pyrophosphato­platinum­(II) (cHxAm-2), and the related compound diisopropyl­amine­dihydrogen­pyrophosphato­platinum­(II) (iPrAm-2) using our protocols.

Further, we attempted to oxidize this series of compounds to their analogous *cis*,*trans*-dicyclo­alkyl­amine­dihydroxo­dihydrogen­pyrophosphato­platinum­(IV) complexes by adding 30% hydrogen peroxide (1 mL) to our reaction mixtures at the end of the initial incubation period and then allowing them to incubate for up to an additional 3 h at 40 °C, following the procedure of Mishur and Bose.^19^ Following this incubation, solutions were concentrated and the pH was lowered to ∼1 by addition of nitric acid to protonate the platinum complexes. Unfortunately, we were unable isolate any Pt­(IV) complexes in this manner, although ^31^P NMR spectroscopy suggests that sodium *cis*,*trans*-dicyclo­butyl­amine­dihydroxo­pyro­phosphato­platinate­(IV) (cBuAm-4) was successfully prepared in situ, as evidenced by a singlet at 2.59 ppm (Figure S10). This peak has clear satellites due to coupling with the spin 1/2 Pt-195 nucleus with a coupling constant of 26.5 Hz and is shifted downfield from the signal observed in cBuAm-2 as expected due to the greater deshielding effect of Pt­(IV). This is consistent with Pt–P coupling constants for other Pt­(IV)-pyro­phosphato complexes which range from 16 Hz for *cis*,*trans*-Pt​(NH_3_)_2_(OH)_2_(P_2_O_7_)^2–^ to 25 Hz for *trans*-Pt​(ethylene­diamine)​(OH)_2_(P_2_O_7_)^2–^ and *trans*-Pt​(diamino­cyclo­hexane)​(OH)_2_(P_2_O_7_)^2–^.^19^ It should be noted that these satellites are not typically observed for Pt­(II) species, due to chemical shift anisotropy. We also tried to isolate cBuAm-4 by dissolving purified cBuAm-2 in dilute base (NaOH­(aq), pH 8), adding peroxide, and evaporating the solution to dryness following incubation. This resulted in the decomposition of the product into a bright yellow solid that was not further characterized.

### Physical Properties in Aqueous Solution

The aqueous stability of cBuAm-2 at various pH points was examined by preparing 10 mM aqueous solutions and recording daily ^31^P NMR spectra. Like other phosphaplatins,^19^ the pyrophosphate moiety on cBuAm-2 was observed to be stable in aqueous solution for at least 1 week at pH 7, as evidenced by the lack of changes in the ^31^P NMR spectrum during this time (Figure 3a). Lowering the pH to 5.4 resulted in spectral changes within 24 h, with a new peak appearing at 15.01 ppm that grew in intensity over time (Figure 3b). A peak at −9.73 ppm also appeared by day 4 and persisted throughout the experiment (Figure S11). After 5 days, the intensity of the ^31^P signal from cBuAm-2 was reduced to 92% of the total peak area. At pH 4.3, both new peaks were apparent in the ^31^P NMR spectrum within 24 h, and they slowly increased in intensity over 7 days (Figure 3c). After 5 days at pH 4.3, the original compound was found to make up 73% of the total ^31^P NMR signal. This was reduced further to 71% of the total signal by day seven. These results are consistent with observations from Mishur (for dach-2)^19^ and Curci (for *cis*-1,4-cyclo­hexane­diamine­pyro­phosphato­platinate­(II)),^8^ and suggests that phosphaplatins may exhibit reduced aqueous stability in the tumor microenvironment (TME), where the extracellular pH ranges from 5.5 to 7.^25^ To simulate physiological conditions, an additional experiment was performed using 10 mM cBuAm-2 in a 1X PBS buffer (137 mM NaCl, 2.7 mM KCl, 8 mM Na_2_HPO_4_, and 2 mM KH_2_PO_4_), pH 7.38, and holding the solution at 37 °C for the duration of the experiment. The ratio of the integrated areas of peaks from cBuAm-2 and the phosphate peak in the ^31^P NMR spectrum was monitored daily over 7 days (expected ratio is 2:1). During this time, the observed integral for the phosphate peak ranged from 47 to 57% of the signal from cBuAm-2, with no clear trend. No additional peaks were observed at any point. At the end of the experiment, a modest pH increase was observed, with a final measured pH of 7.64.

**Figure 3:**
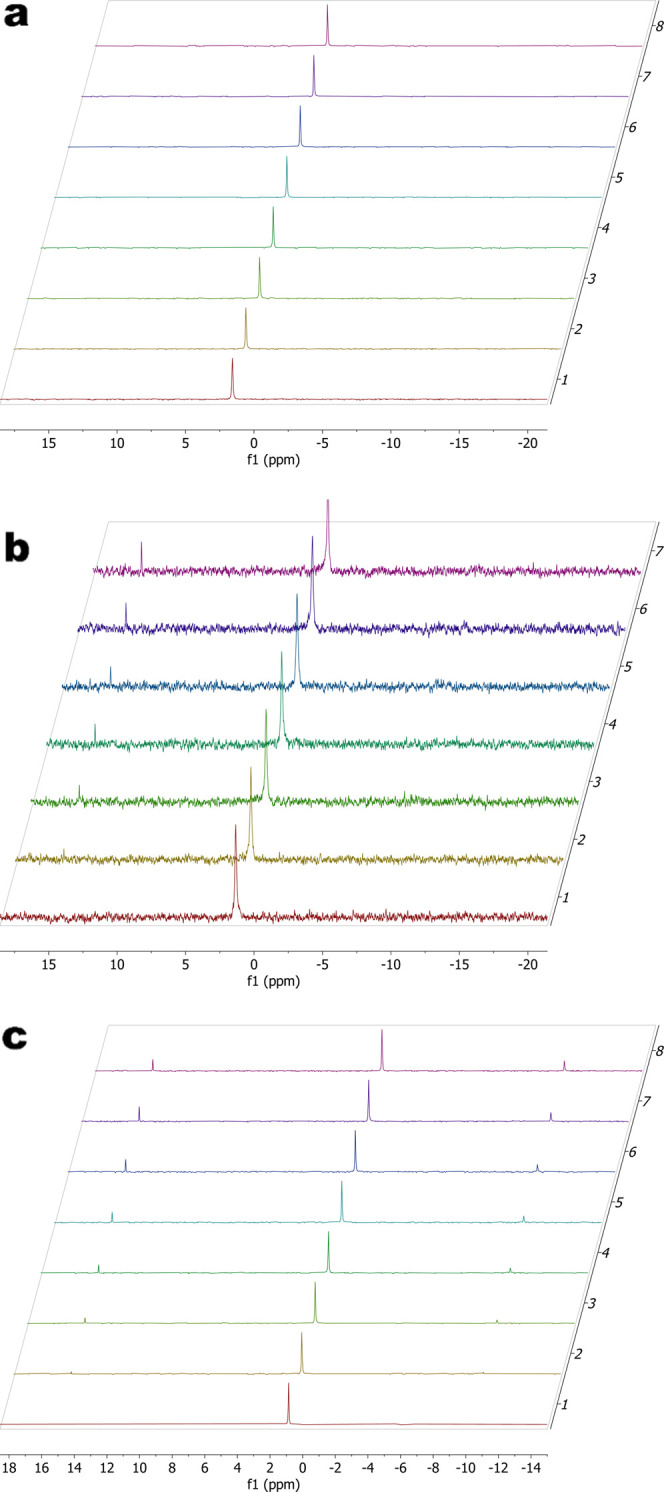
^31^P NMR spectra of an aqueous solution of cBuAm-2 were taken daily to assess its aqueous stability at various pHs. Stacked plots are from day 0 (bottom) through day 7 (top). At pH 7 (a), no spectral changes are observed. At pH 4.3 (c), a new peak at 15 ppm appeared within 24 h, accompanied by an additional peak at −9.7 ppm. Similar changes were observed at an intermediate pH of 5.3 (b), though the peak at −9.7 ppm was obscured by noise. Increasing the number of scans reveals this peak (Figure S11).

The singlets at 15 and −9.73 ppm in the ^31^P NMR spectrum can be explained by deligation of the pyrophosphate ligand, followed by the formation of a dinuclear platinum complex containing a tetradentate pyrophosphate bridging ligand. As there is no spectral evidence for a monodentate pyrophosphate complex, it is postulated that this species is short-lived compared to the NMR time scale, and that formation of this complex is the rate-limiting step, consistent with the previously proposed mechanism (Figure 4a). At the end of the experiment at pH 4, the NMR tube was saved, and plate-like crystals slowly grew out of the solution over the next several weeks. These crystals were analyzed by X-ray crystallography and confirmed to belong to a dinuclear platinum complex with a tetradentate pyrophosphate bridging ligand: μ-pyro­phosphato­tetrakis­(cyclo­butyl­amine)­diplatinum­(II) (Figure 4b).

**Figure 4:**
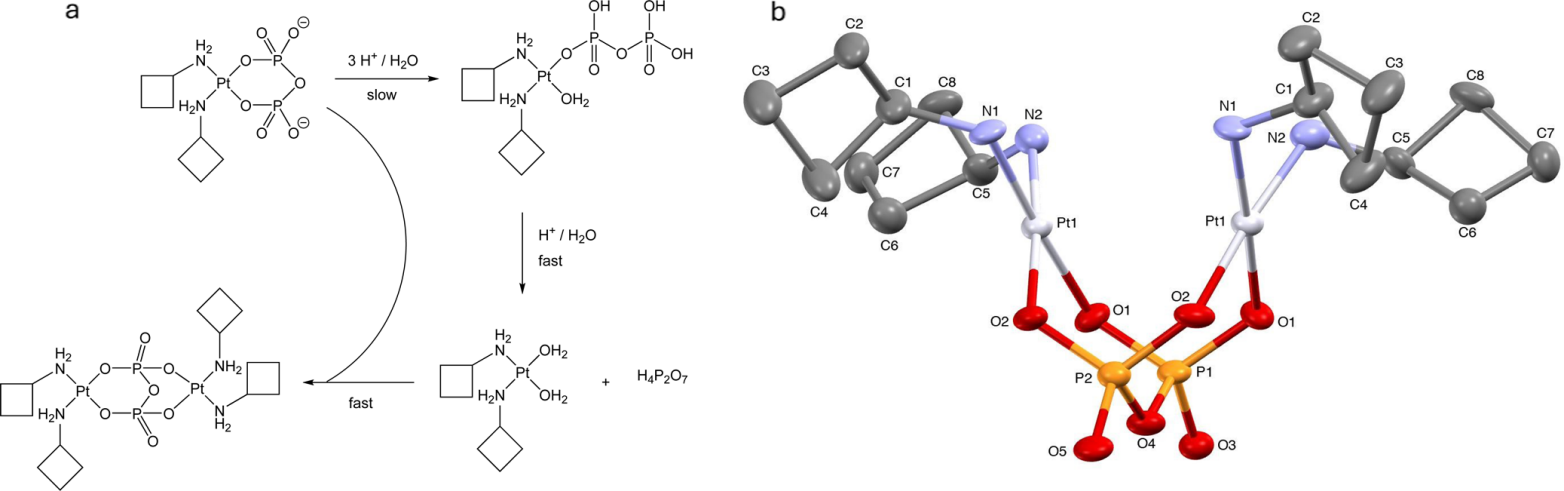
(a) Proposed mechanism for formation of dinuclear platinum complex; (b) X-ray crystal structure of Pt dimer that resulted from slow evaporation of a dilute aqueous solution of cBuAm-2.

In the solid state, the molecule rests on a mirror plane. The cyclobutyl group was treated to three-dimensional anisotropic displacement rigid-group restraints. A cocrystallized water molecule was treated to idealized geometry based on initial locations of the H atoms from the electron density difference map with *U*_iso_ equal to 1.5 *U*_eq_ of the attached oxygen atom. The water molecule was located disordered in two positions with a refined site occupancy ration of 75/25. Non-hydrogen atoms were refined with anisotropic displacement parameters. Hydrogen atoms, other than the water H atoms, were treated as idealized contributions with geometrically calculated positions and with *U*_iso_ equal to 1.2 *U*_eq_ of the attached carbon atom. Atomic scattering factors are contained in the SHELXTL program library.^16^ The structure has been deposited at the Cambridge Structural Database under CCDC 2302238, DOI: 10.5517/ccdc.csd.cc2h8nr6. Crystal data and refinement details are summarized in Table 1.

Additionally, we investigated whether cBuAm-2 is stable in the presence of pyrophosphatase, an enzyme found in most living cells, that breaks down pyrophosphate in vivo, resulting in the formation of two phosphate ions.^26^ When inorganic pyrophosphate was treated with an excess of pyrophosphatase, a rapid reaction occurred, as evidenced by the complete disappearance of the pyrophosphate ^31^P NMR signal within the first 10 min of the reaction and the emergence of a new signal at 2.5 ppm, which can be assigned to orthophosphate ion, PO_4_^3–^ (Figure S12). However, when cBuAm-2 was incubated with pyrophosphatase under the same conditions, no spectral changes were observed up to 18 h (Figure S13). The reaction was not monitored past that point. Our results, consistent with the results of Mishur and Bose,^19^ show that once the pyrophosphate moiety is bound to platinum in a bidentate fashion, this enzyme is unable to act on it. Aqueous stability studies at different pH and in the presence and absence of pyrophosphatase for cPnAm-2 were not performed, due to limited quantities. However, it is not expected that this compound would have different aqueous chemistry than other known phosphaplatins.

Phosphaplatins are diprotic, and the protonation state of these compounds influences the ^31^P chemical shift of nearby phosphorus nuclei. We determined p*K*_a_ values for cBuAm-2 and cPnAm-2 by plotting the ^31^P chemical shift versus pH and fitting the curve to the equation for a diprotic acid (Figure 5). The experimentally determined p*K*_a_ values, along with values for other representative phosphaplatins, are provided in Table 2. As expected, cPnAm-2 is less acidic than cBuAm-2, due to a greater inductive effect as previously discussed. We did not determine p*K*_a_ values for cPrAm-2, though it would be expected to be slightly more acidic than cBuAm-2.

**Figure 5:**
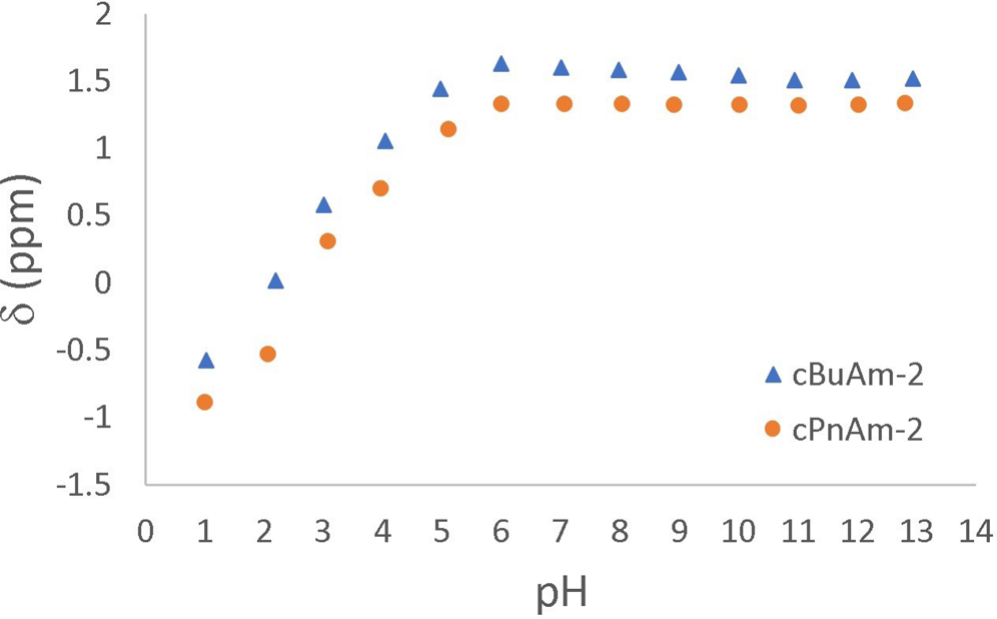
^31^P NMR chemical shift vs pH of cBuAm-2 (p*K*_a1_ = 2.18, p*K*_a2_ = 4.09) and cPnAm-2 (p*K*_a1_ = 2.54, p*K*_a2_ = 4.69).

**Table 2:** Experimentally Determined p*K*_a_ Values Are Provided for the Two Compounds Described in This Paper and Compared to Other Representative Pt­(II)-Pyrophosphato Complexes^a^

compound	N donor ligand(s)	p*K*_a1_	p*K*_a2_	reference
cBuAm-2	cyclo­butylamine	2.18	4.09	this paper
cPnAm-2	cyclo­pentylamine	2.54	4.69	this paper
1,2-dach-2	1,2-diamino­cyclo­hexane	2.6	4.4	Mishur^19^
1,3-dach-2	1,3-diamino­cyclo­hexane	2.7^a^	5.0^a^	Barbanente^9^
1,4-dach-2	1,4-diamino­cyclo­hexane	2.4^a^	4.9^a^	Curci^8^
1,2-dachex-2	*trans*-1,2-diamino-4-cyclo­hexene	2.4^a^	4.6^a^	Barbanente^9^
en-2	ethylene­diamine	2.2	4.4	Mishur^19^
am-2	ammine	2.9	4.7	Mishur^19^

a All compounds consist of a Pt­(II) nucleus bound to pyrophosphate in a bidentate fashion, and the ligand(s) listed in the second column. 1,2-Diamino­cyclo­hexane, 1,4-diamino­cyclo­hexane, and ethylene­diamine bind in a bidentate manner. All other listed ligands bind monodentate, with a cis geometry for the *N*-donor ligands.

b Values were determined in D_2_O, and a constant of 0.4 was applied to these values to correct for the fact that observed pH is lower in D_2_O than in water. All other values were determined in H_2_O, using an internal capillary of D_2_O to provide the deuterium lock for NMR.

### Binding of Phosphaplatins to Nucleotides

The biological mechanism of action for cisplatin is well-established to involve the formation of DNA adducts by binding to purine bases. While early reports of phosphaplatins suggested that they do not form covalent bonds with DNA bases,^7^ Curci et al. have shown that at least one member of this class of molecules, 1,4-diamino­cyclo­hexane­pyro­phosphato­platinum­(II), can react with 5′-guanine monophosphate in vitro.^8^ Further, Prachařová demonstrated that, contrary to initial findings, DNA-binding also occurs for RRD2, an enantiomer of dach-2, and that these DNA lesions contribute to the antiproliferative effect of the compound.^27^ As part of this study, we examined the ability of cBuAm-2 and cPnAm-2 to bind to AMP and dGMP. For comparison, we also examined cisplatin and racemic dach-2. Reaction mixtures containing 2 mM Pt and 5 mM of either AMP or dGMP were monitored by proton and ^31^P NMR for up to 77 h.

Cisplatin forms covalent bonds with dGMP in as little as 6 h by binding to the N7 position on the purine ring. This is evidenced by ^1^H NMR, where a decrease of the signal from the H8 proton of guanine (8.00 ppm) is observed, along with the appearance of new peaks at 8.40 and 8.44 ppm (Supplemental Figure S14). We assign the peak at 8.44 ppm to the H8 proton of a Pt-bound species, in which one of the chloro-groups on cisplatin has been replaced by coordination to guanine. A downfield chemical shift is observed due to the strong deshielding effect of the Pt­(II) nucleus. This peak appears to decrease in intensity after 6 h, and the peak at 8.40 ppm is observed to increase throughout the reaction. We postulate that this peak corresponds to a species in which both chloro-groups on cisplatin have been replaced, resulting in a bis-dGMP platinum complex. After 24 h an unassigned triplet appears at 6.08 ppm (not shown). This peak grows throughout the reaction, with a corresponding decrease in the intensity of the triplet at 6.15 ppm corresponding to the deoxyribose ring. At the end of the reaction, a ^31^P NMR spectrum was recorded (Supplemental Figure S15). The presence of multiple resonances provides direct evidence that a reaction occurred, as dGMP contains a single phosphorus nucleus.

Cisplatin also forms covalent bonds to AMP within the first 6 h of the reaction, though the new features in the ^1^H NMR spectrum at 6 h were barely above the noise threshold (Supplemental Figure S16). This suggests that the initial binding to adenine is kinetically slower than binding to guanine, as new features are prominent in the ^1^H NMR spectrum of the reaction of cisplatin with guanine at 6 h, and is consistent with the fact that the in reactions with DNA, cisplatin forms approximately twice as many *cis*-[Pt­(NH3)_2_{d­(GpG)}] adducts as *cis*-[Pt­(NH3)_2_{d­(ApG)}] adducts.^28^ These peaks increased in intensity over 48 h, at the end of which a ^31^P NMR spectrum was acquired (Supplemental Figures S17 and S18). As with the reaction with dGMP, multiple resonances were observed in the ^31^P NMR spectrum at the end of 48 h.

Conversely, when dach-2 was incubated with dGMP, no spectral changes were observed within 48 h (Supplemental Figures S19 and S20). However, dach-2 reacted with AMP within the first 24 h, as evidenced by gross spectral changes in both the ^1^H and ^31^P NMR spectra (Supplemental Figures S21 and S22). Similarly, cBuAm-2 appears to react more rapidly with AMP than dGMP, as changes in the ^1^H NMR spectrum appear within 24 h for the reaction of cBuAm-2 with AMP (Figure 6), but not until 48 h for the reaction of cBuAm-2 with dGMP (Supplemental Figure S23). Further, in the reaction of cBuAm-2 with dGMP no spectral changes could be seen in the ^31^P NMR spectrum in 48 h (data not shown). For cPnAm-2, faint spectral features corresponding to new species can be observed within 24 h of reaction with either AMP or dGMP (Supplemental Figures S24–S27). We assume that these species are similar in nature to those formed by cisplatin, representing mono- and *cis*-bifunctional Pt-nucleotide adducts. However, it is also possible that cis/trans isomerization of the di­cyclo­alkyl­amine­platinum­(II) complex occurs following loss of pyrophosphate, as *cis*-di­chloro­dicyclo­butyl­amine­platinum­(II) has been shown to easily convert to the *trans* isomer under certain conditions.^29^ No attempt was made to characterize the products.

**Figure 6:**
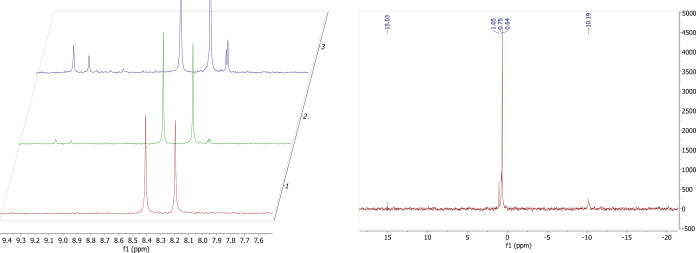
(Left) ^1^H NMR spectra of the reaction of cBuAm-2 with AMP after 0, 24, 48 h (bottom to top). New features can be seen starting at 24 h and increasing in intensity throughout the reaction. (Right) ^31^P NMR spectrum of the reaction of cBuAm-2 with AMP following 49 h shows multiple resonances, including a peak at −10.19 ppm (free pyrophosphate). Additionally, a peak at −15 ppm is observed, which we assign to a dinuclear platinum species.

The reaction with cisplatin was only followed for 24 h, at which point a 86 ± 5% decay of the H8 proton signal from dGMP was observed (Supplemental Figure S28). However, in the same time period, a decrease of only 61 ± 2% of the AMP signals (H8 + H2) was observed (AVG ± STDEV of 2 trials). This is consistent with our findings that cisplatin binds to dGMP more quickly than AMP and is expected for cisplatin.

Because they bind to nucleotides more slowly than cisplatin, phosphaplatins were allowed to react with the nucleotide mixture for 8 days. At the end of 8 days, the reaction with dach-2 resulted in a decrease of 62 ± 10% in the signal from dGMP, and a decrease of 22 ± 6% in the signal from AMP (AVG ± STDEV of 3 trials, Supplemental Figure S29). The ratio of dGMP adducts to dAMP adducts is twice that observed for cisplatin, indicating an even stronger preference for binding to guanine.

When this experiment was repeated with cBuAm-2, we observed a decrease of 37% in the dGMP NMR signal, and no noticeable decrease in the AMP NMR signals, which implies that cBuAm-2 is significantly less capable of binding to AMP than either cisplatin or dach-2 (Supplemental Figure S30). Taken together, this data suggests that the nature of the nonleaving groups plays a significant role in determining the type of DNA adducts that are formed.

The fact that the nucleotide adducts formed by dach-2 favor guanine over adenine in a 2.8:1 ratio, along with our findings that dach-2 reacts with AMP more rapidly than it does with dGMP, implies that initial Pt-AMP adducts may be converted to more stable Pt-GMP adducts. However, we caution against overinterpretation of these results for several reasons: (1) NMR spectroscopy is inherently less sensitive than other techniques, requiring analyte concentrations of approximately 1–2 mg/mL, meaning low-abundance species may be missed, (2) several of the adduct peaks were small compared to the level of noise in the spectra, which could affect proper integration, and (3) spectra were acquired in water, which is the largest peak in the spectra; however, because the integrated region (approximately 7.5–9 ppm) is far from the water peak (4.7 ppm), baseline effects should be minimal, and care was taken to correct the baseline as needed in the region of interest. As a point of note, a 5 mM solution of cisplatin should be able to bind to a maximum of 10 mM nucleotide, assuming bis-adduct formation. Since both nucleotides were present at a concentration of 10 mM, this means that the percent decays of the adenine and guanine peaks when added together should not exceed 100%. Nonetheless, our results are interesting, and it would be informative to repeat this experiment using a more sensitive analytical technique, such as LC–MS.

### Kinetics of Reactions of Di­cyclo­alkyl­amine­pyro­phosphato­platinum­(II) Compounds with Cysteine and Glutathione

It has been established that only ∼1% of intracellular cisplatin bonds to nuclear DNA.^30^ The remainder forms covalent bonds to various peptides and proteins, primarily those containing cysteine residues, driven by favorable soft–soft acid–base interactions with Pt­(II). Therefore, we decided to probe the kinetics of the reaction of our compounds with both cysteine and glutathione (GSH), a tripeptide comprised of cysteine, glycine, and glutamic acid. GSH is present in cells at concentrations ranging from 1 to 10 mM,^31^ and bonding to GSH is a known cisplatin deactivation pathway.^3^

All reactions were performed under pseudo-first-order conditions, where the concentration of the thiol was at least 20 times higher than the concentration of platinum. Reactions were followed by ^31^P NMR, and pseudo first-order rate constants (*k*_obs_) were determined by plotting the natural log of the normalized NMR signal versus time and fitting the resulting plot to the equation for a line, *y* = *mx* + *b*, using Microsoft Excel, where −*m* represents *k*_obs_ = *k*_1_[thiol] (Figure 7a). All experiments were performed in at least triplicate. For cPnAm-2, only the reaction with cysteine was performed, due to limited quantities of the platinum complex. Results are shown in Table 3.

**Figure 7:**
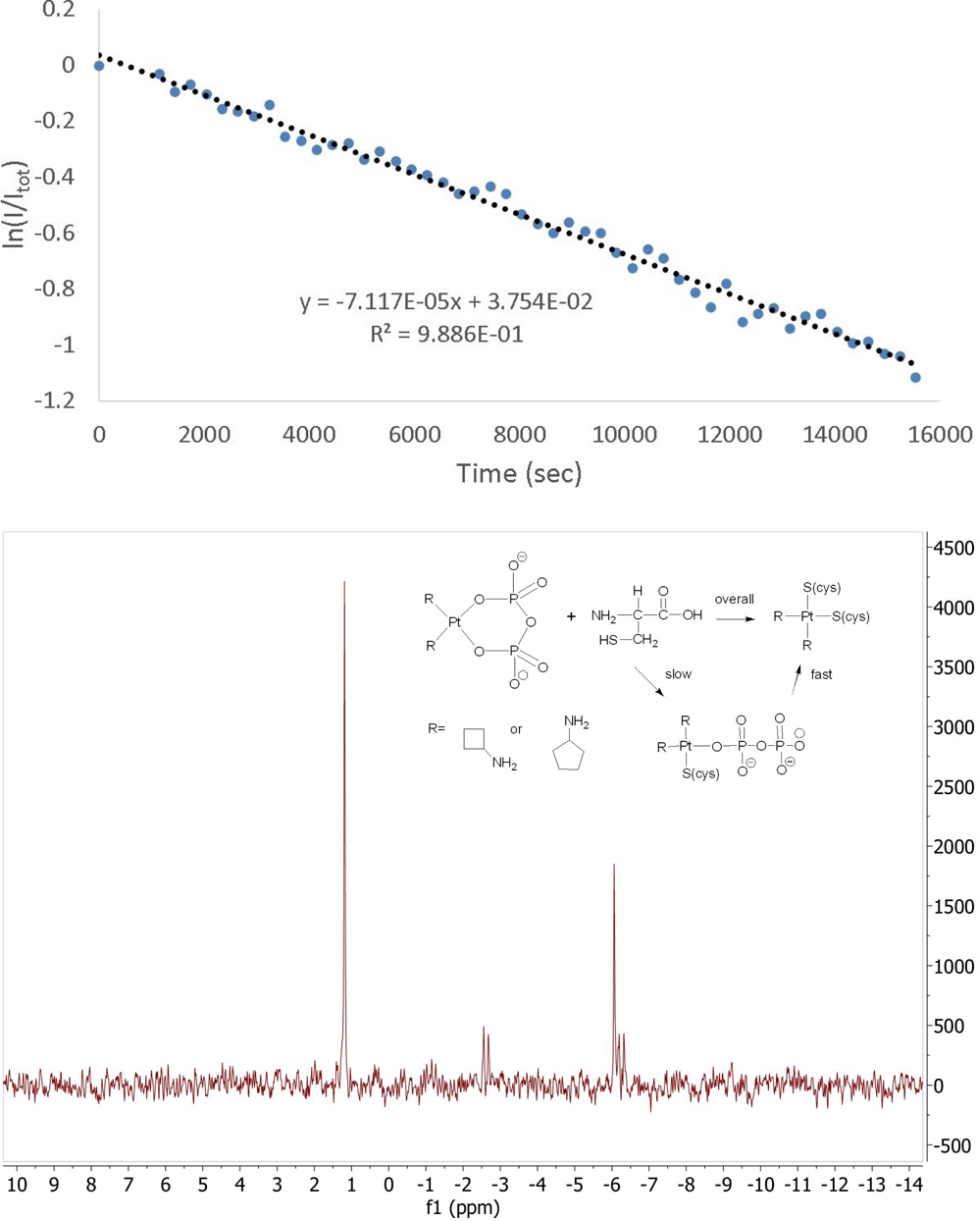
(Top) Pseudo-first-order plot of the reaction of cPnAm-2 with cysteine. The negative slope of the plot gives the pseudo first-order rate constant. (Bottom) ^31^P NMR spectrum acquired during the reaction of cPnAm-2 with cysteine shows two doublets that can be assigned to a monodentate platinum-pyrophosphato compound. The proposed reaction scheme is shown in the inset.

**Table 3:** Observed and Calculated First-Order Rate Constants for the Reactions of Phosphaplatins with Cysteine and Glutathione

	*k*_obs_, s^–1^	*k*_1_, M^–1^ s^–1^
cBuAm-2 + GSH	5.28 ± 0.98 × 10^–6^	8.80 ± 1.36 × 10^–5^
cBuAm-2 + cys	2.64 ± 0.40 × 10^–5^	4.41 ± 0.66 × 10^–4^
cPnAm-2 + cys	6.32 ± 2.27 × 10^–5^	1.05 ± 0.40 × 10^–4^

For reactions involving cPnAm-2, a transient species was observed, as evidenced by a pair of doublets in the ^31^P NMR spectrum (*J*_PP_ = 48 Hz, Figure 7b). This is due to the pyrophosphate group deligating at one end, resulting in a short-lived monodentate platinum-pyrophosphato species (Figure 7b, inset). We have also observed the formation of a transient monodentate species in reactions with dach-2 (data not published); however, in reactions with cBuAm-2, this species is too short-lived to be observed on the NMR time scale. Values for *k*_2_ were unable to be determined from the data.

### Viability Study

Relative IC_50_ values for cBuAm-2, cPnAm-2, and RRD2/PT112 were determined in a human breast cancer cell line and a human lung adenocarcinoma cell line using a MTT cell viability assay. Cells were also treated with cisplatin, a well-established cytotoxic agent, as a means to validate the method. However, a shorter treatment time was used for cisplatin (48 h vs 72 h for phosphaplatins), precluding any direct comparisons. Cell viability data is provided in Supplemental Tables S1–S4 and experimentally determined IC_50_ values are given in Table 4. The relative IC_50_ value for RRD2, an established phosphaplatin which is currently undergoing clinical trials, was 1.3 μM, indicating that this compound is very potent, as expected. Newly synthesized *cis*-di­cyclo­alkyl­amine­pyrophosphato­platinates were significantly less effective compared to RRD2 in the same cell lines. One possible explanation is isomerization to *trans* species following loss of pyrophosphate, as previously discussed. However, as square planar complexes typically react via associative mechanisms, we find this possibility unlikely.

**Table 4:** Experimental IC_50_ Values of Various Phosphaplatins Determined by Using an MTT Assay in Human Lung Cancer (A549) and Human Breast Cancer (MDA-MB-231) Cell Lines^a^

	A549	MDA-MB-231
cBuAm-2	44.58	98.01
cPnAm-2	36.27	85.25
RRD2	1.344	3.071
cDDP	6.811	14.44

a Values reflect 72 h treatment with phosphaplatins, or 48 h treatment with cisplatin. All concentrations are given in μM and represent the average of three biological replicates. cDDP = cisplatin.

The cyclopentylamine compound had lower IC_50_ values than the cyclobutylamine compound in both cell lines in which it was tested, which is consistent with the findings by Braddock et al. that *cis*-dichloro­dicyclo­pentlamine­platinum­(II) has a lower ID_90_ than *cis*-di­chloro­dicyclo­butyl­amine­platinum­(II)^13^ (compounds were administered via an intraperitoneal injection to mice that had a plasmacytoma implanted subcutaneously; ID_90_ represents the dose which caused 90% tumor regression). Generally speaking, the nature of both the leaving groups and nonleaving groups on the platinum will affect the molecule’s biological activity. The presence of bulky nonleaving groups, such as the diaminocyclohexane group found in oxaliplatin and PT112, can lead to DNA-adducts that are less easily repaired, improving activity. However, this argument can only be taken so far, as, for example, the potency of *cis*-di­chloro­dicyclo­octyl­amine­platinum­(II) is significantly lower than the potency of *cis-*di­chloro­dicyclo­alkyl­amine­platinum­(II) compounds with smaller cycloalkyl rings.^13^ Further, it is not clear that DNA-binding is the primary mechanism of action for phosphaplatins. It is not yet possible to draw conclusive structure–activity relationships on the limited number of known phosphaplatins, highlighting the need to continue to study and expand this class of molecules.

## Conclusion

In this study, we set out to synthesize a series of Pt-pyrophosphate complexes of the type dicyclo­alkyl­amine­amine­dihydrogen­pyro­phosphato­platinum­(II) using a series of cycloalkylamine ligands C_*n*_H_2*n*–1_–NH_2_, where *n* = 3–6, using a modified version of the method developed by Mishur and Bose.^19^ Two new compounds were isolated, which contained either cyclo­butyl­amine or cyclo­propyl­amine as a ligand. Chemical shifts and acidity constant data both show that increasing the size of the cycloalkylamine ring increases the amount of electron density donated onto the pyrophosphate ligand. Based on IC_50_ data, the cyclopentylamine compound appears to be a better drug candidate than the cyclobutylamine compound, though both displayed IC_50_ values approximately 30*x* higher than the experimental drug RRD2/PT112. Ongoing research aims to further expand on the number of known phosphaplatins, so as to better elucidate structure–activity relationships for this class of molecules.

## Supplementary Material



## Notes

^1^H, ^13^C, and ^31^P NMR spectra and ESI-mass spectra for compounds cBuAm-2 and cPnAm-2 ^31^P NMR spectra for the reaction of *cis*-di­chloro­dicyclo­propyl­amine­platinum­(II) with pyrophosphate and the reaction of cBuAm-2 with hydrogen peroxide, acquired in situ ^31^P NMR spectrum of cBuAm-2 following 4 days of incubation at room temperature, pH 5.4 ^31^P NMR spectra of pyrophosphate and cBuAm-2 following incubation with pyrophosphatase ^1^H NMR spectra of reaction between cisplatin and dGMP at 0, 6, 24, and 48 h ^31^P NMR spectrum of reaction between cisplatin and dGMP after 48 h ^1^H NMR spectra of reaction between cisplatin and AMP at 0, 6, 24, and 48 h ^31^P NMR spectrum of reaction between cisplatin and AMP after 48 h ^1^H NMR spectra of reaction between dach-2 and dGMP at 0, 24, and 48 h ^31^P NMR spectrum of reaction between dach-2 and dGMP after 48 h ^1^H NMR spectra of reaction between dach-2 and AMP at 0, 24, and 48 h ^31^P NMR spectrum of reaction between dach-2 and AMP after 48 h ^1^H NMR spectra of reaction between cBuAm-2 and dGMP at 0, 24, 48, and 77 h ^1^H NMR spectra of reaction between cPnAm-2 and dGMP at 0, 24, 48, and 72 h ^31^P NMR spectrum of reaction between cPnAm-2 and dGMP after 72 h ^1^H NMR spectra of reaction between cPnAm-2 and AMP at 0, 24, 48, and 72 h ^31^P NMR spectrum of reaction between cPnAm-2 and AMP after 72 h ^1^H NMR spectra of the reaction of cisplatin with a mixture of dGMP and AMP at 0 and 24 h ^1^H NMR spectra of the reaction of dach-2 with a mixture of dGMP and AMP at 0 and 8 days ^1^H NMR spectra of the reaction of cBuAm-2 with a mixture of dGMP and AMP at 0 and 8 days MTT cell viability assay data for A549 and MDA-MB-231 cell lines treated with phosphaplatins (PDF)

## ∥

University of Delaware, Department of Chemistry and Biochemistry, Newark, DE 19716, U.S.A
