# Supplementary material for: Synthesis, Characterization, and Cytotoxicity of Dicyclo­alkyl­amine­pyrophosphato­platinum​(II) Complexes
Source: ACS Omega. 2026 Feb 10;11(7):11233–44. doi: 10.1021/acsomega.5c07828 (PMC12947200; doi:10.1021/acsomega.5c07828)
Supplement: Supplementary file 1 [file ao5c07828_si_001.pdf]

Synthesis, characterization, and cytotoxicity of dicycloalkylaminepyrophosphatoplatinum(II) complexes

Dianne M. Wagner,[1,‡] Dieu Huyen My Nguyen,[1,‡,†] Emily McHenry,[2] Lanise A. Brown,[1] Taylor Lindholm,[1] Sarita S. Yadav,[1] Glenn P. A. Yap,[3] Michael J. Toneff,[2] and Robert J. Mishur[1,*]
[1]Widener University, Department of Chemistry, One University Pl, Chester, PA 19013, USA
[2]Widener University, Department of Biology, One University Pl, Chester, PA 19013, USA
[3]University of Delaware, Department of Chemistry and Biochemistry, Newark, DE 19716, USA

[†]Current address University of Delaware, Department of Chemistry and Biochemistry, Newark, DE 19716, USA
[*]Author to whom correspondence should be addressed. Email: rjmishur@widener.edu
[‡]Authors contributed equally

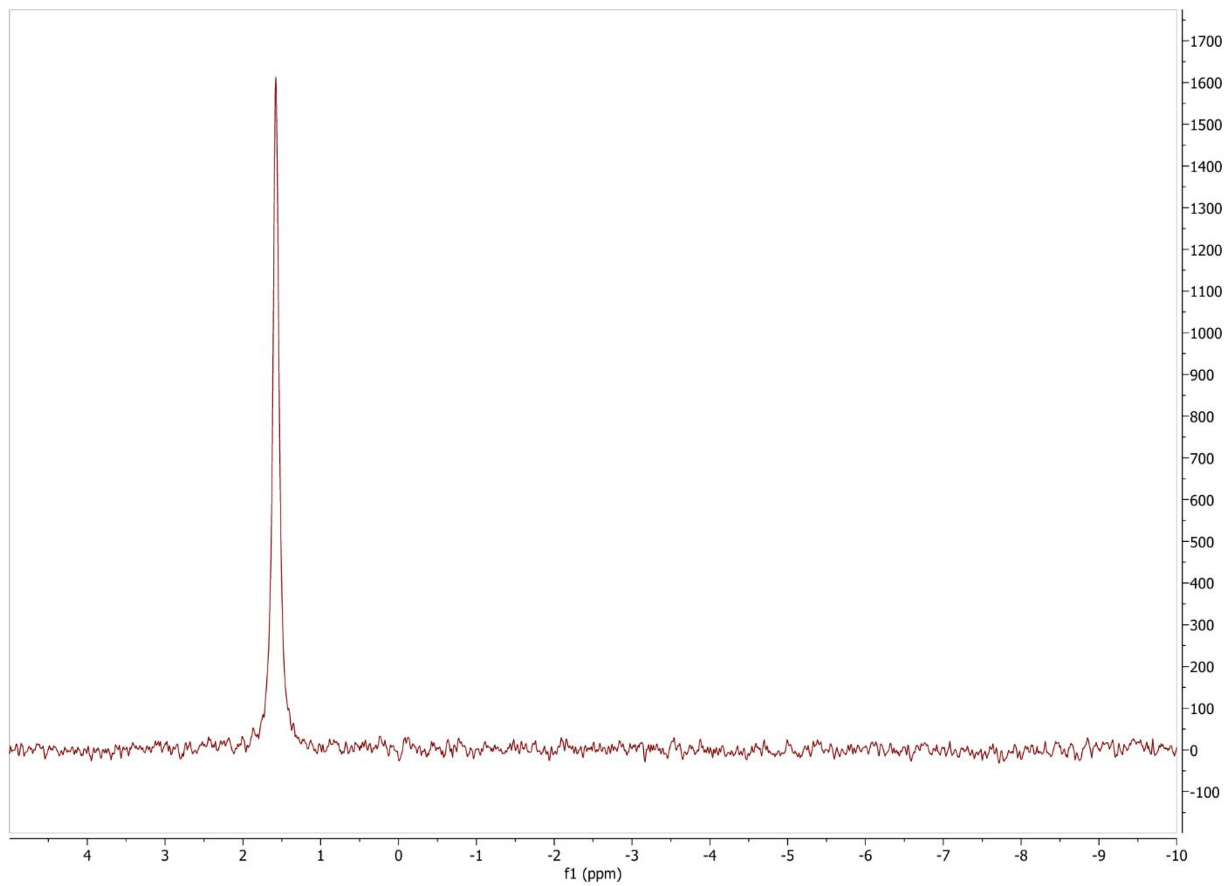

Figure S1: Phosphorus-31 NMR spectrum of cBuAm-2

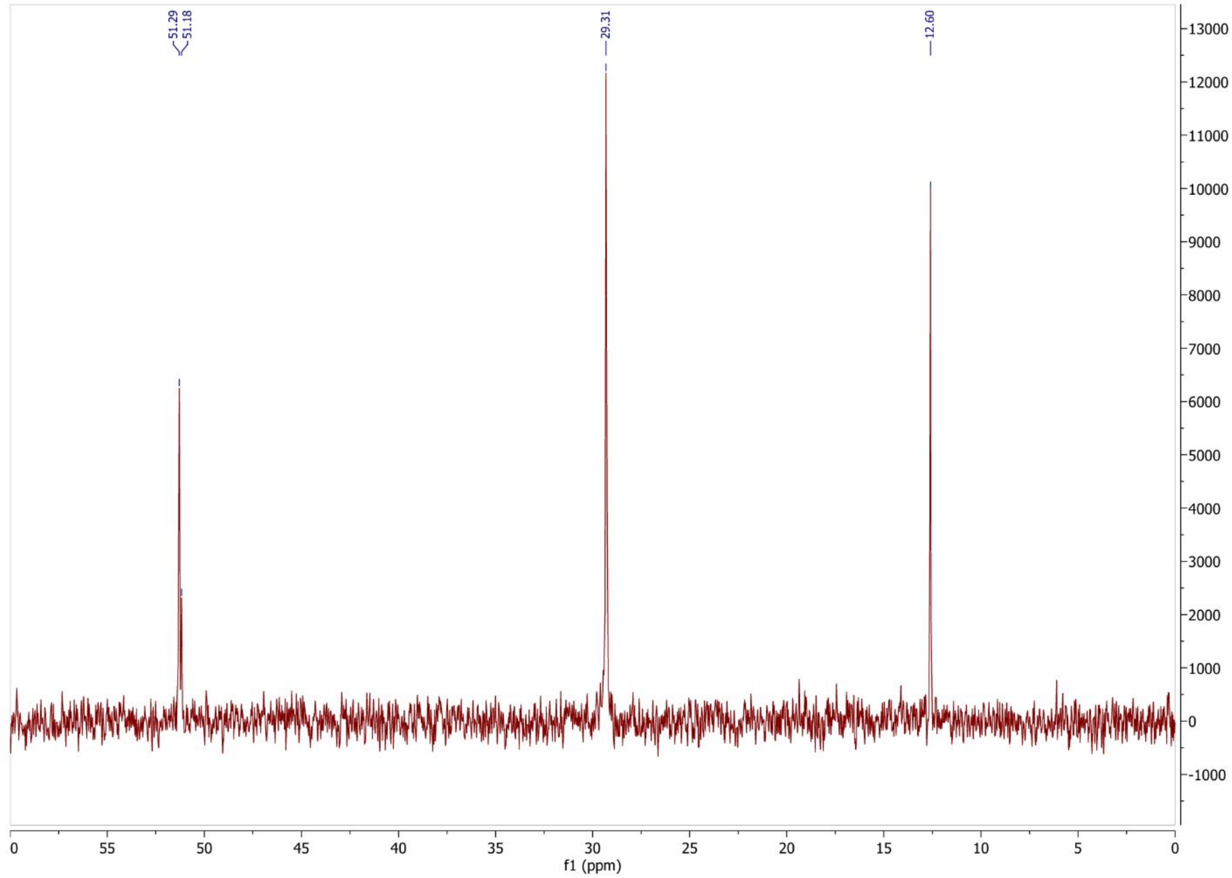

Figure S2: Carbon-13 NMR spectrum of cBuAm-2

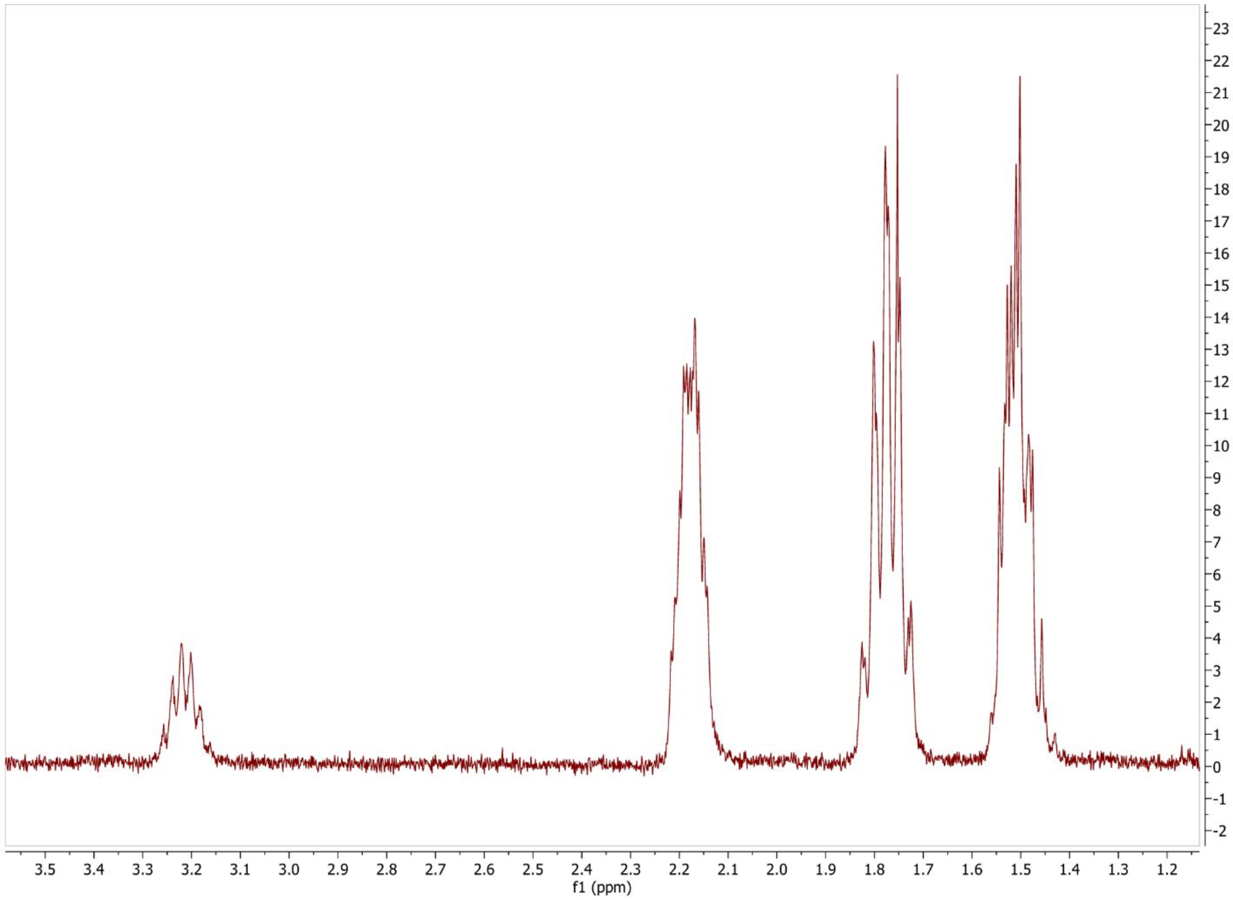

Figure S3: Proton NMR spectrum of cBuAm-2

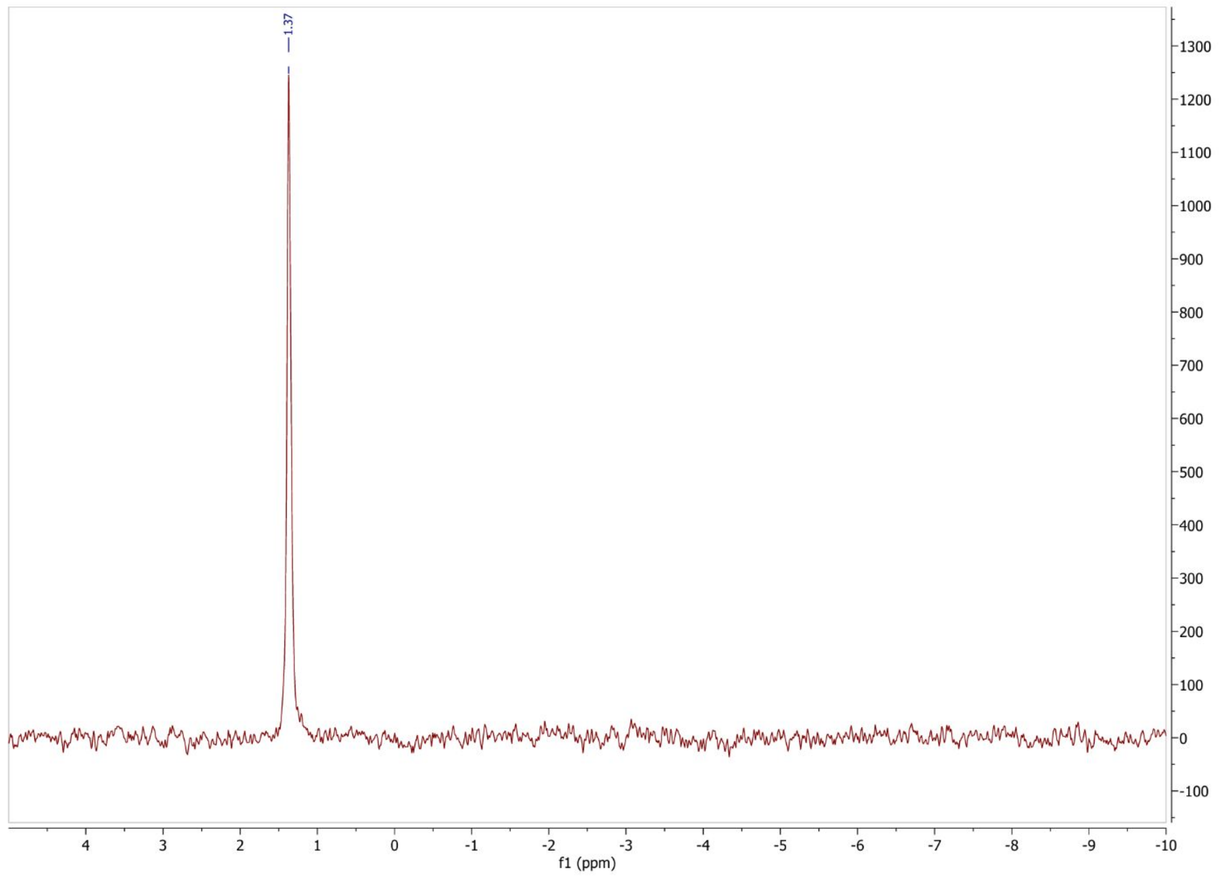

Figure S4: Phosphorus-31 NMR spectrum of cPnAm-2

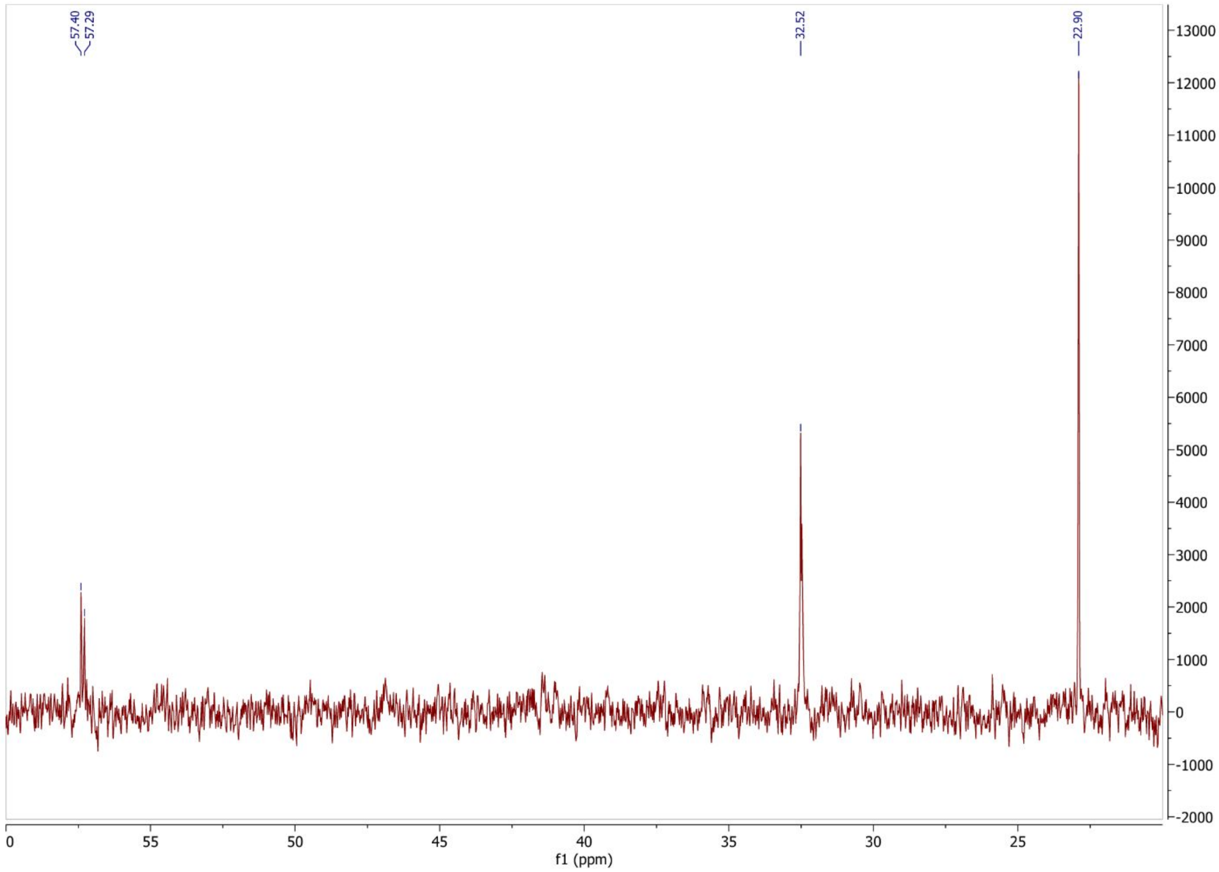

Figure S5: Carbon-13 NMR spectrum of cPnAm-2. There is an additional unresolved singlet at 32.47 ppm.

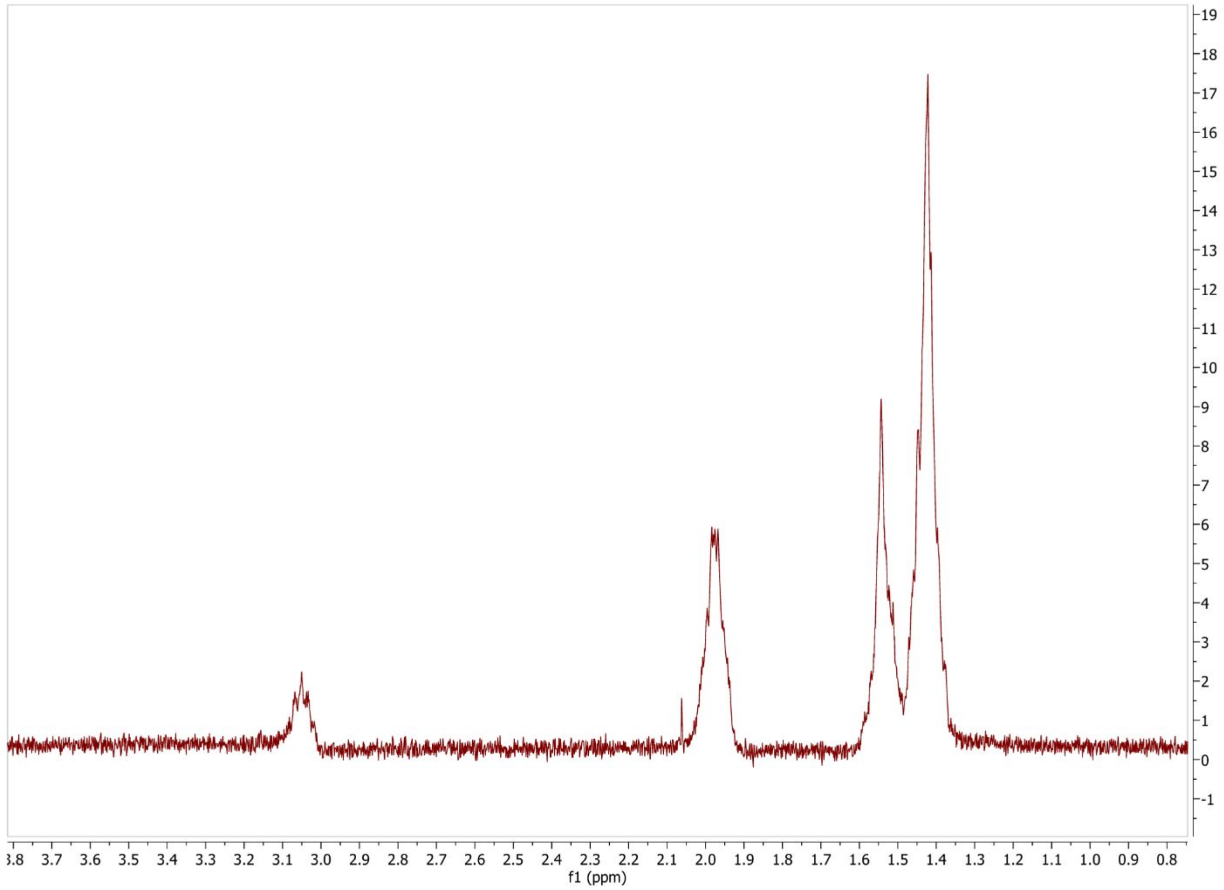

Figure S6: Proton NMR spectrum of cPnAm-2

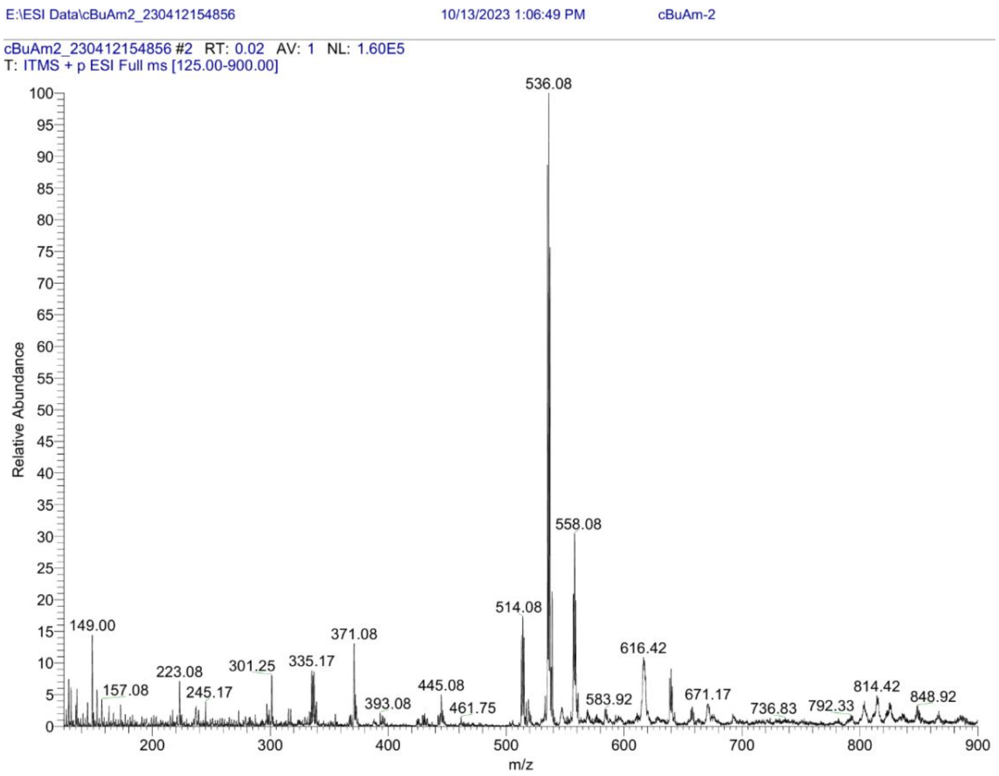

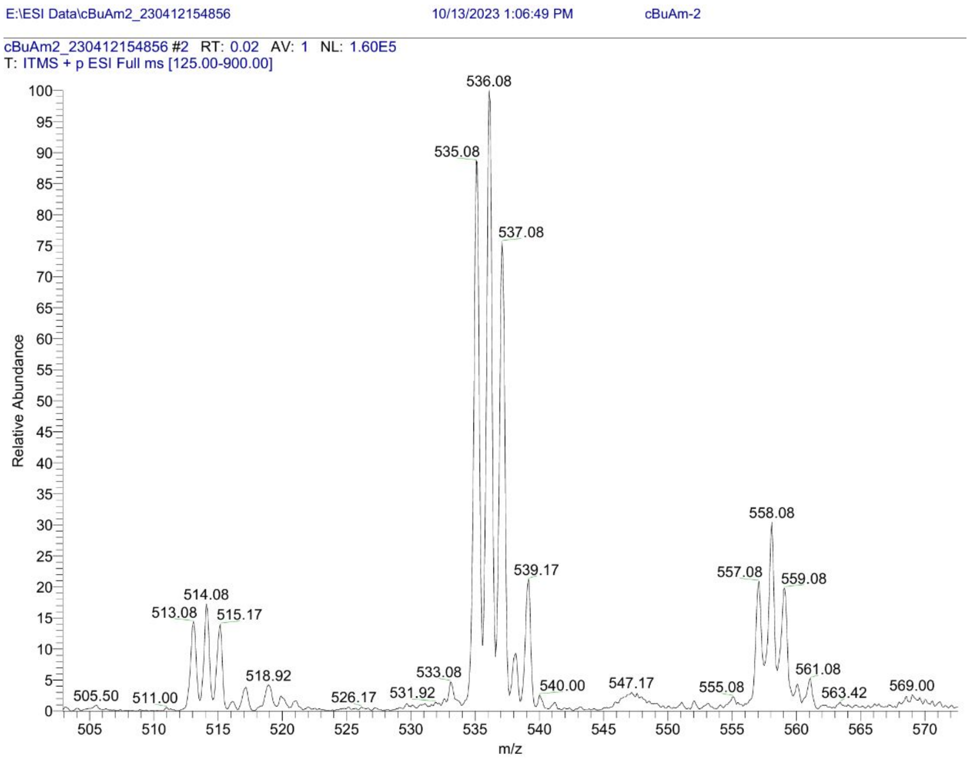

Figure S7: Top - ESI-MS spectrum of cBuAm-2 acquired in positive ion mode. Bottom - zoomed spectrum showing peaks corresponding to $[M+H]^+$ (*m/z* 514), $[M+Na]^+$ (*m/z* 536), and $[M+2Na-H]^+$ (*m/z* 558).

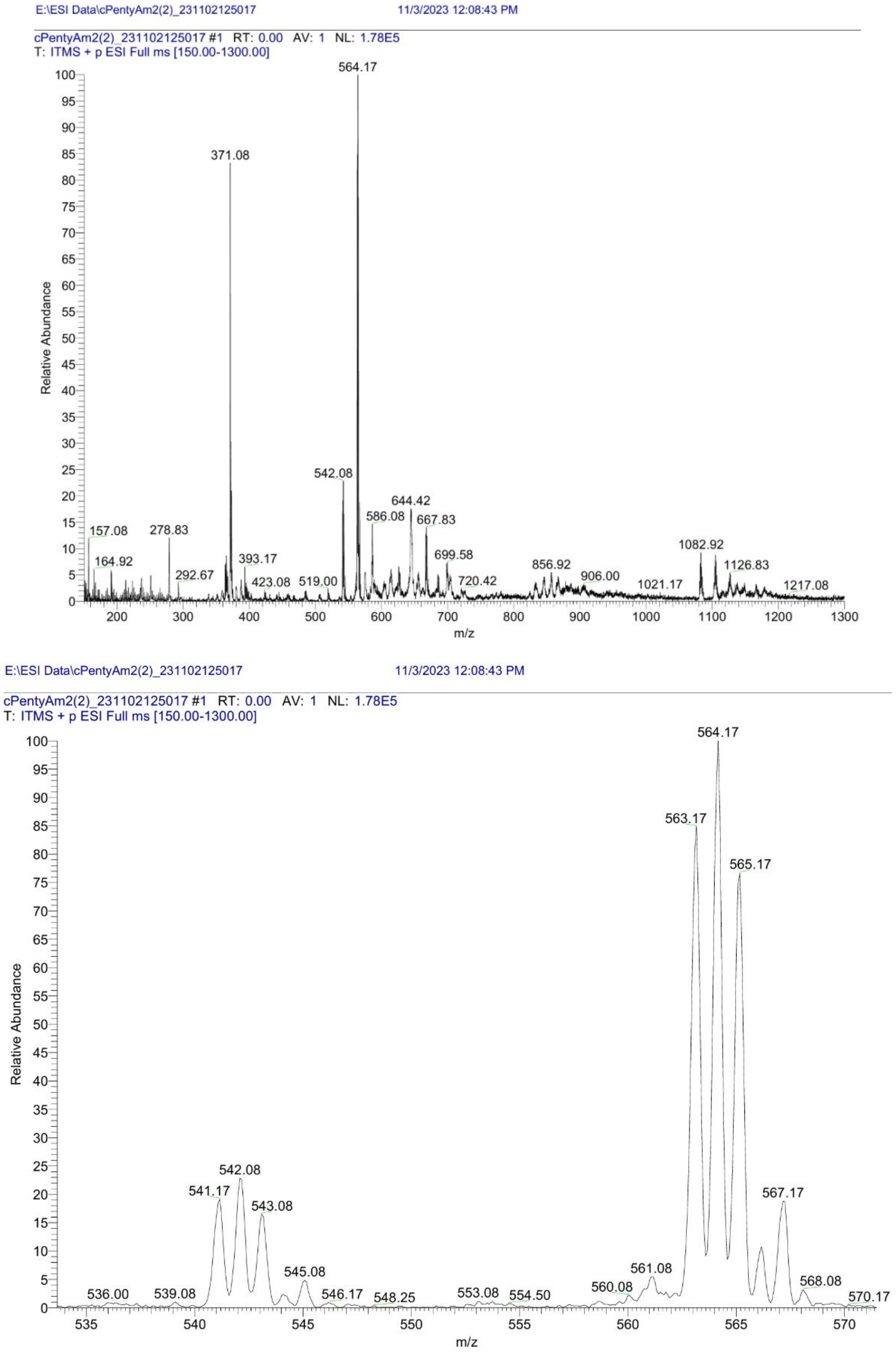

Figure S8: Top - ESI-MS spectrum of cPnAm-2 acquired in positive ion mode. Bottom - zoomed spectrum showing peaks corresponding to $[M+H]^+$ (*m/z* 542) and $[M+Na]^+$ (*m/z* 564).

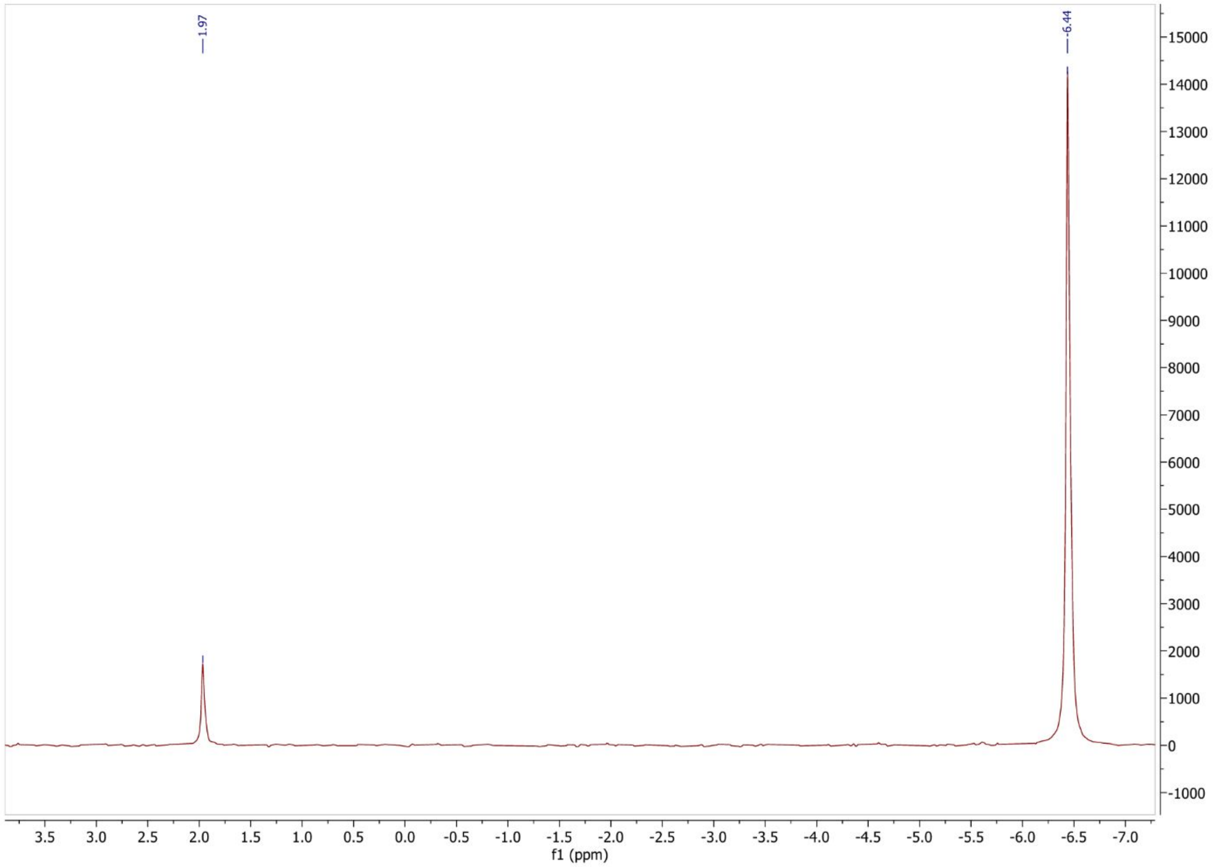

Figure S9: The phosphorus-31 NMR spectrum at the end of reaction of *cis*-dichlorodicyclopropylamineplatinum(II) and pyrophosphate suggests that cPrAm-2 was generated *in situ* (1.97 ppm), though we were unable to isolate it from solution.

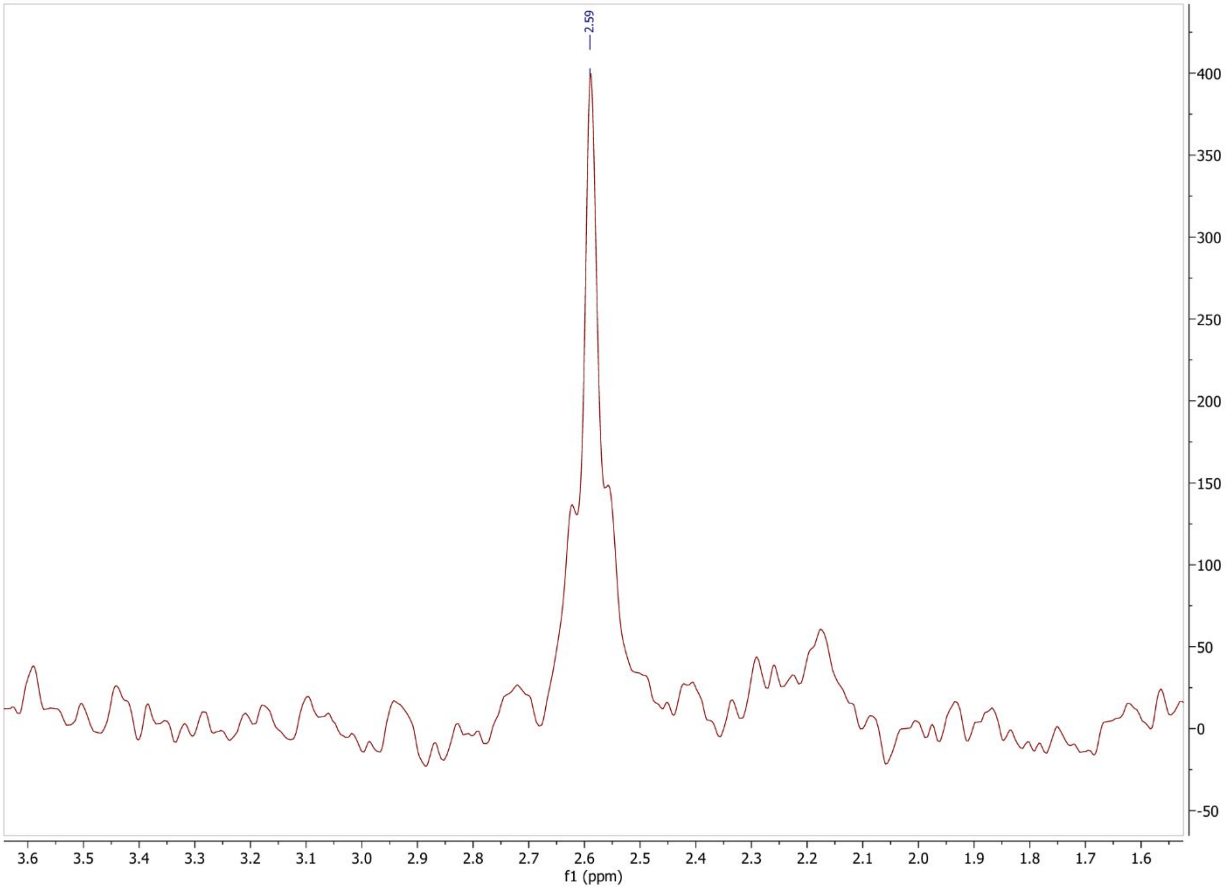

Figure S10: The reaction of cBuAm-2 with hydrogen peroxide in solution results in the disappearance of the cBuAm-2 peak and the appearance of a new peak further downfield in the ^{31}P NMR spectrum. This, along with the appearance of satellites, strongly suggests a +4 oxidation state for platinum. This compound was not isolated from solution.

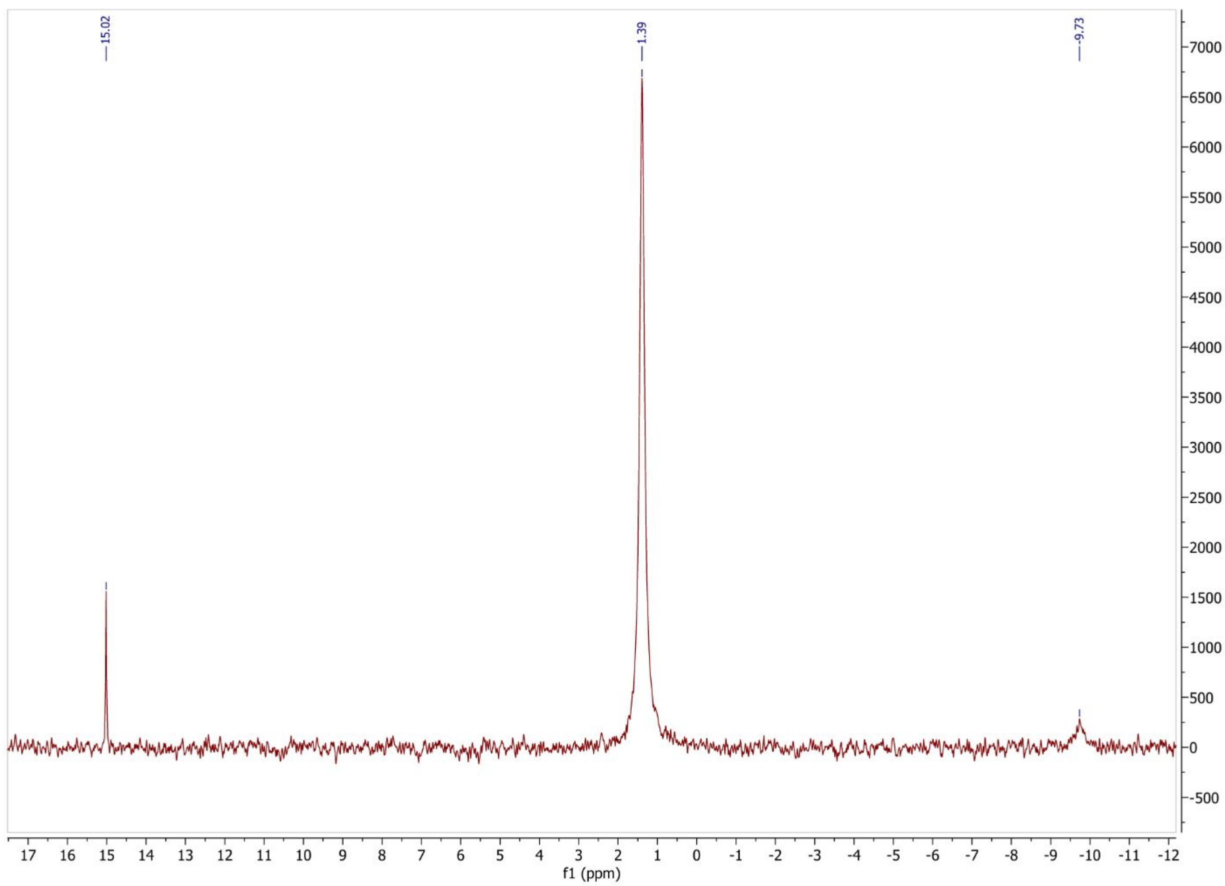

Figure S11: Phosphorus-31 NMR spectrum of 10 mM cBuAm-2, pH 5.4, after 4 days. The peak at -9.73 ppm is assigned to the pyrophosphate ion, and the peak at 15.02 ppm represents a new platinum species that formed in solution during this time.

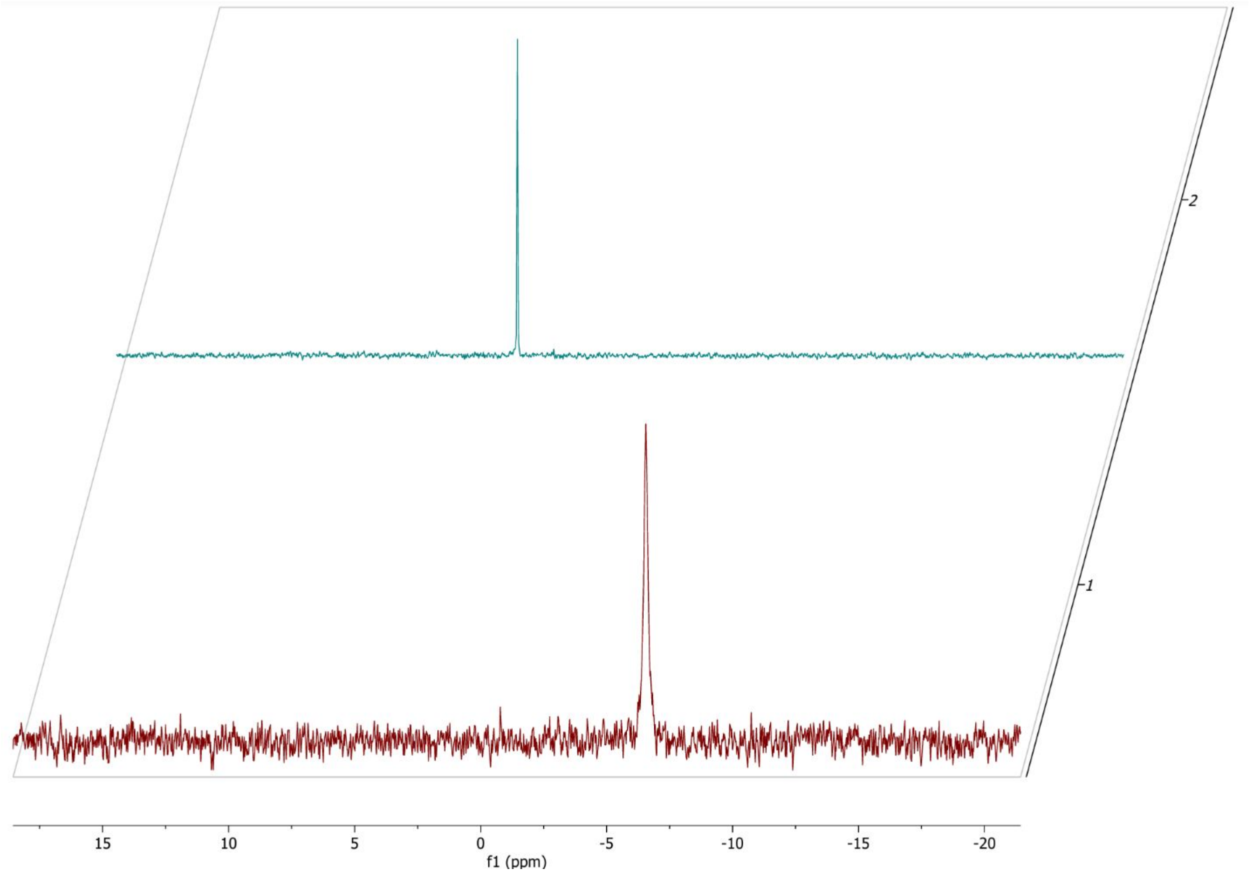

Figure S12: Phosphorus NMR spectrum of pyrophosphate + pyrophosphatase; bottom 0 hr, top 40 min. Within the first 40 minutes of the reaction, the pyrophosphate peak is completely degraded, and a new peak appears at 2.5 ppm, corresponding to the orthophosphate ion.

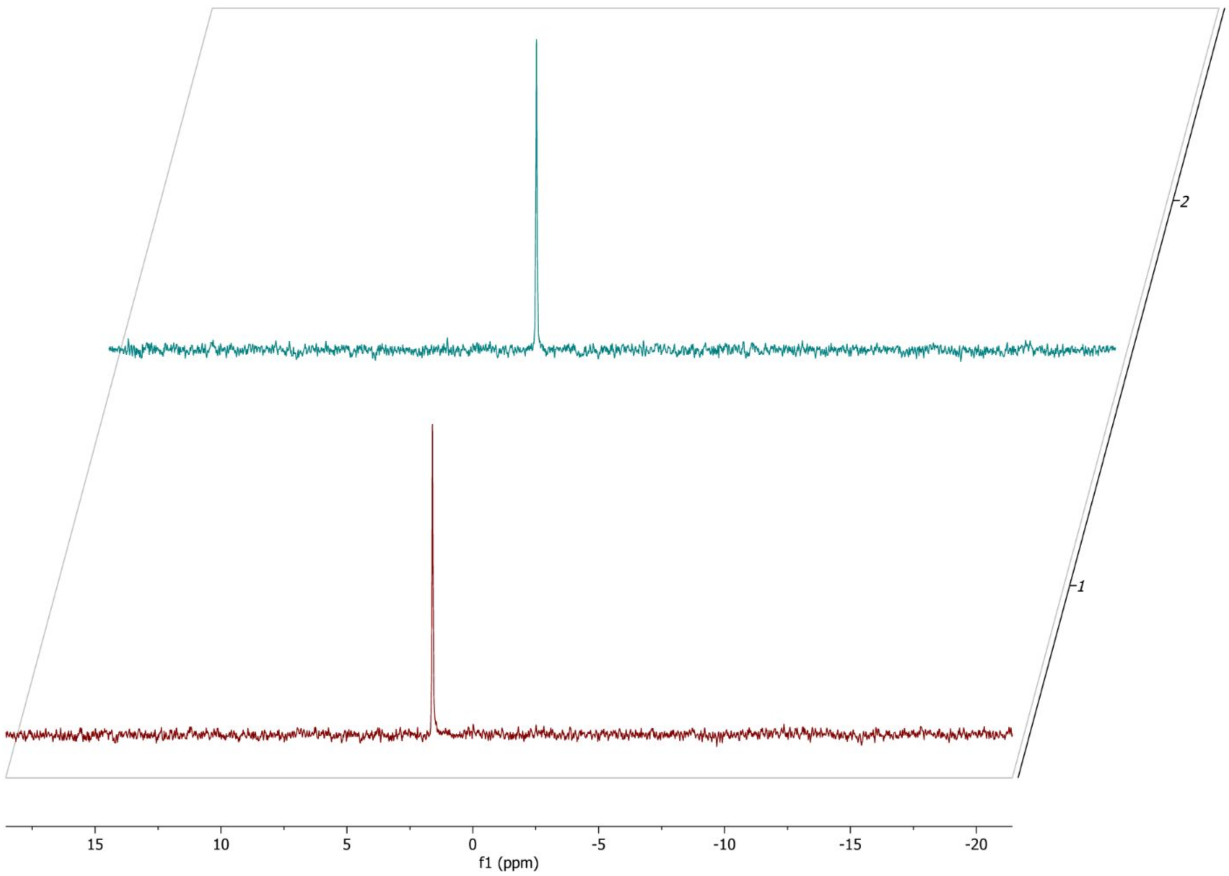

Figure S13: Phosphorus-31 NMR spectrum of cBuAm-2 + pyrophosphatase; bottom 0 hr, top 18 hr. No spectral changes were observed during this time.

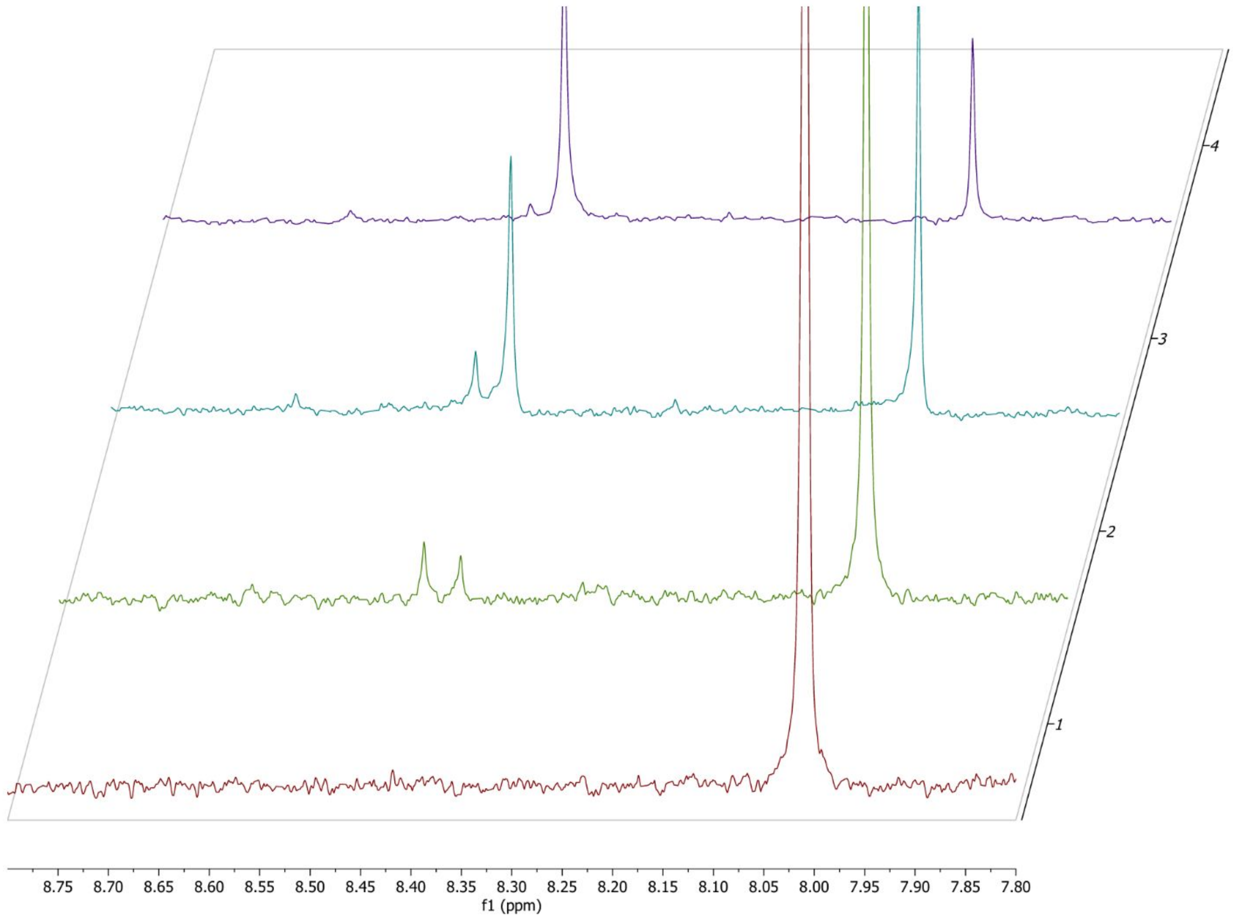

Figure S14: Proton NMR spectra of the reaction of cisplatin with dGMP after 0, 6, 24, and 48 hours (bottom to top). The large peak at 8.0 ppm corresponds to the H8 proton on guanine. The appearance of additional peaks downfield confirms coordination of the guanine to cisplatin.

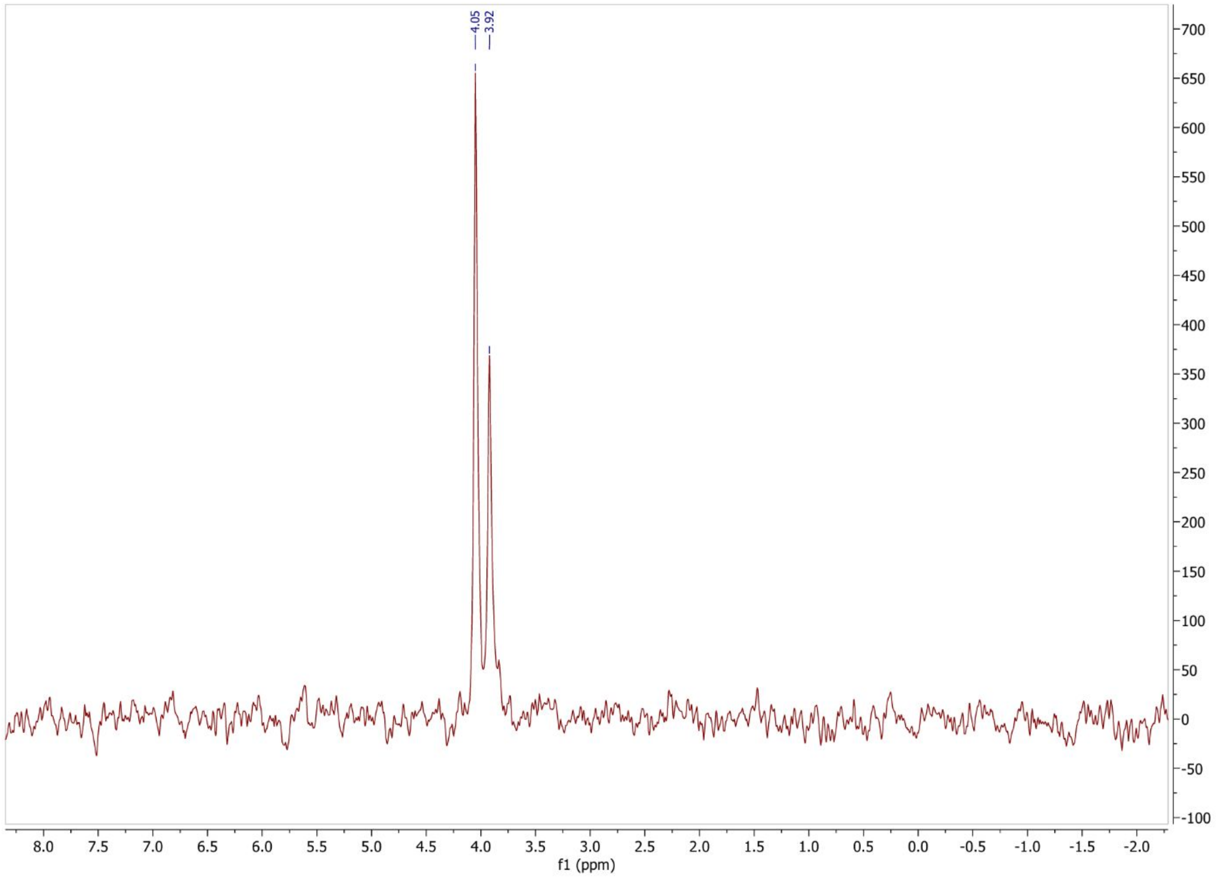

Figure S15: The phosphorus-31 NMR spectrum of the reaction of cisplatin with dGMP following 48 hours clearly shows multiple resonances indicating a reaction had occurred.

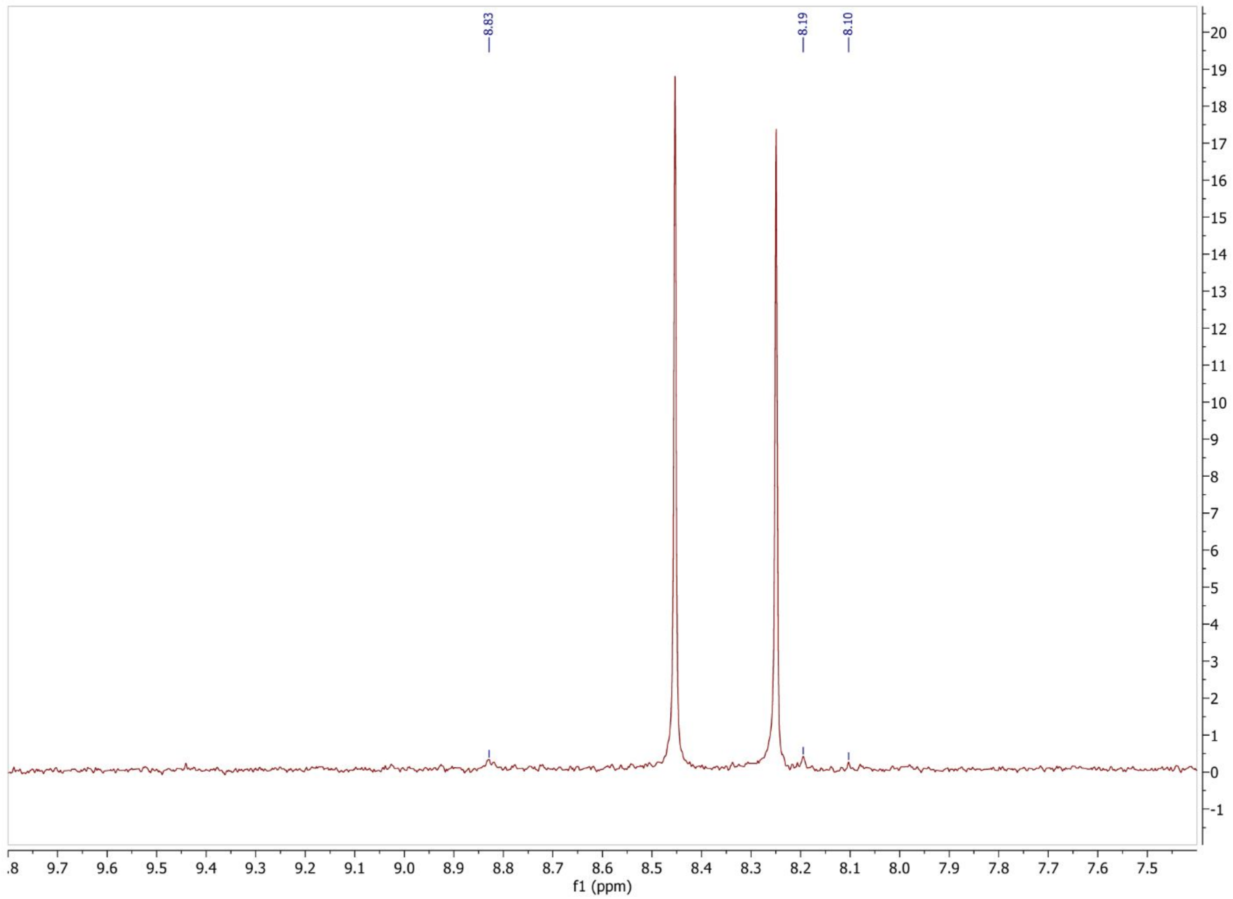

Figure S16: Proton NMR spectra of the reaction of cisplatin with AMP after 6 hours. The peaks at 8.26 and 8.47 ppm correspond to the H8 and H2 protons on adenine, respectively. New peaks can be seen emerging at 8.10, 8.19, and 8.88 ppm, which grow throughout the course of the reaction.

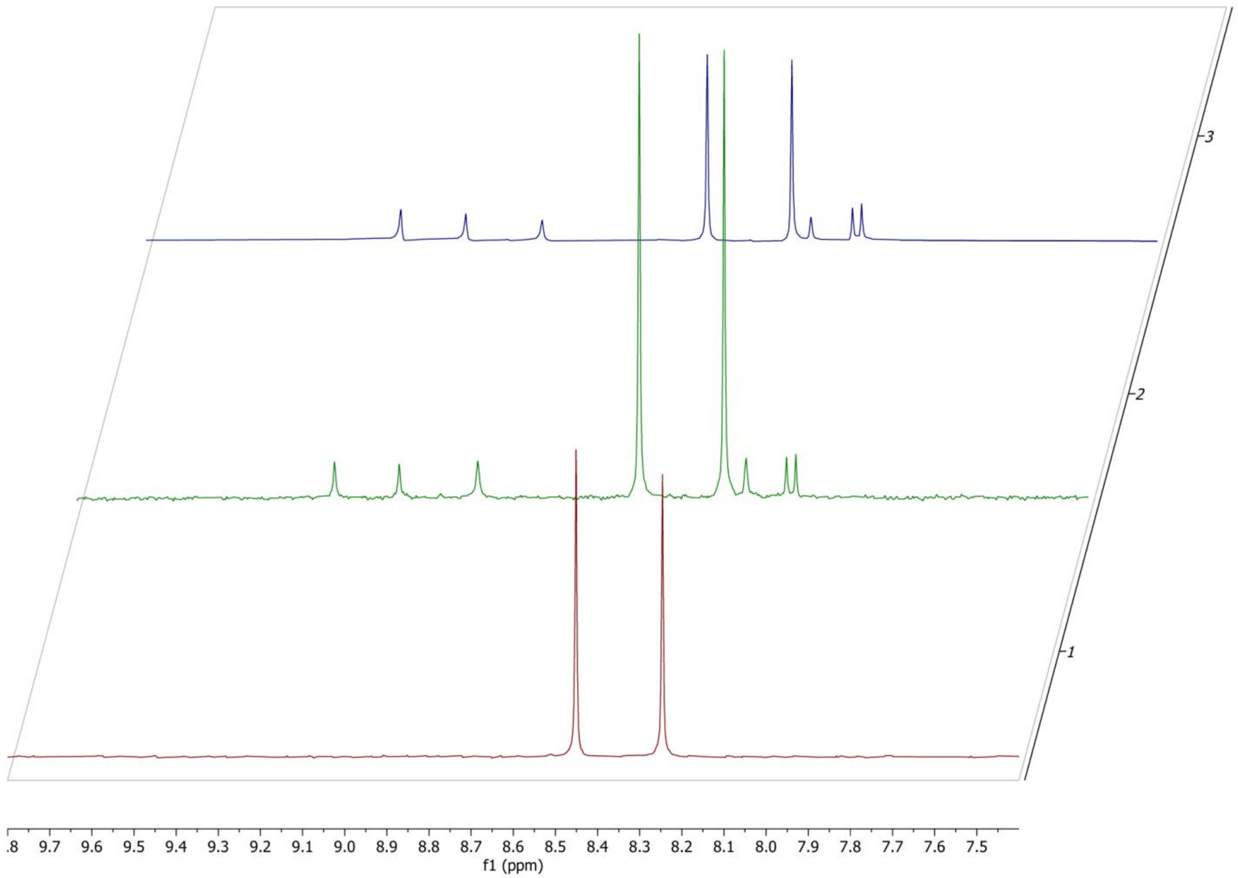

Figure S17: Proton NMR spectra of the reaction of cisplatin with AMP after 0, 24, and 48 hours (bottom to top). The peaks at 8.26 and 8.47 ppm correspond to the H8 and H2 protons on adenine, respectively. The appearance of additional peaks downfield confirms coordination to cisplatin. No attempt was made to assign these peaks.

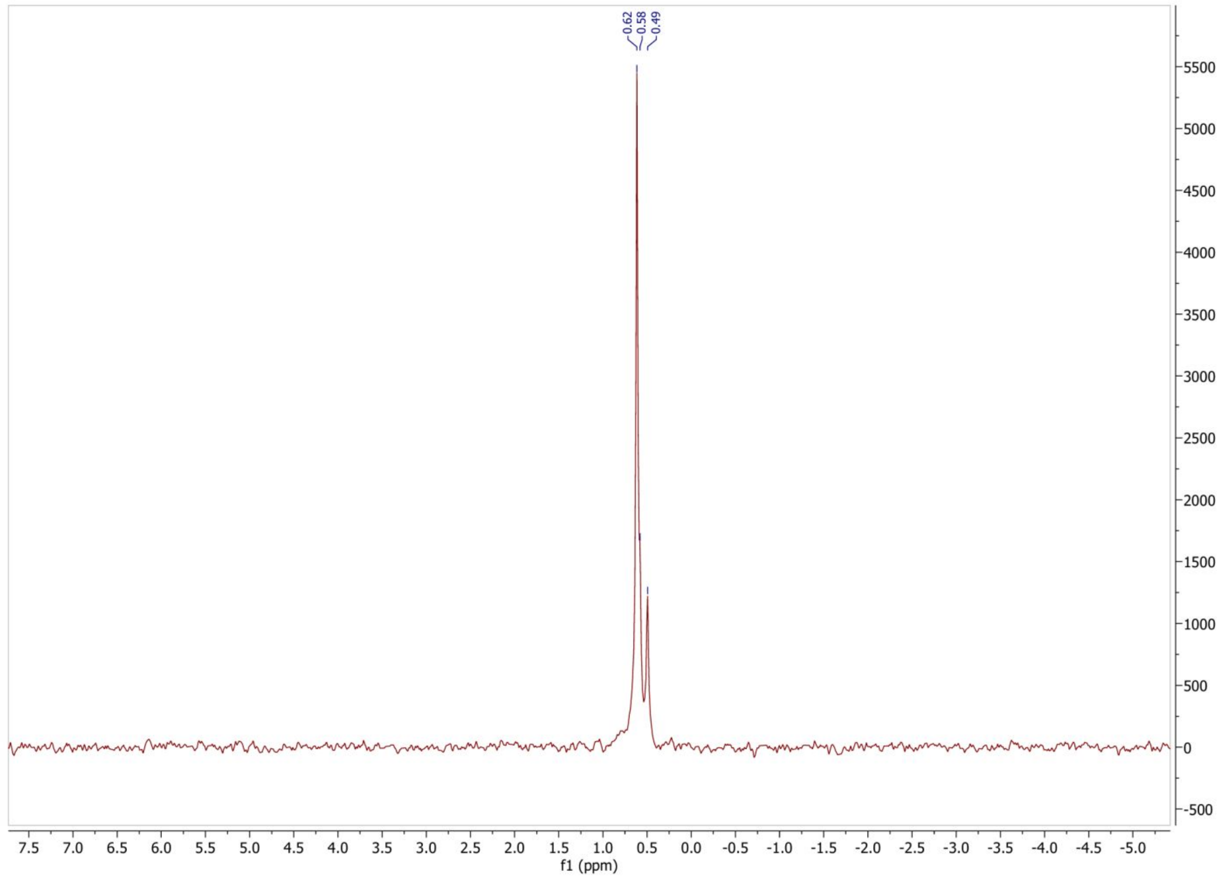

Figure S18: The phosphorus-31 NMR spectrum of the reaction of cisplatin with AMP following 48 hours shows multiple resonances, indicating a reaction had occurred.

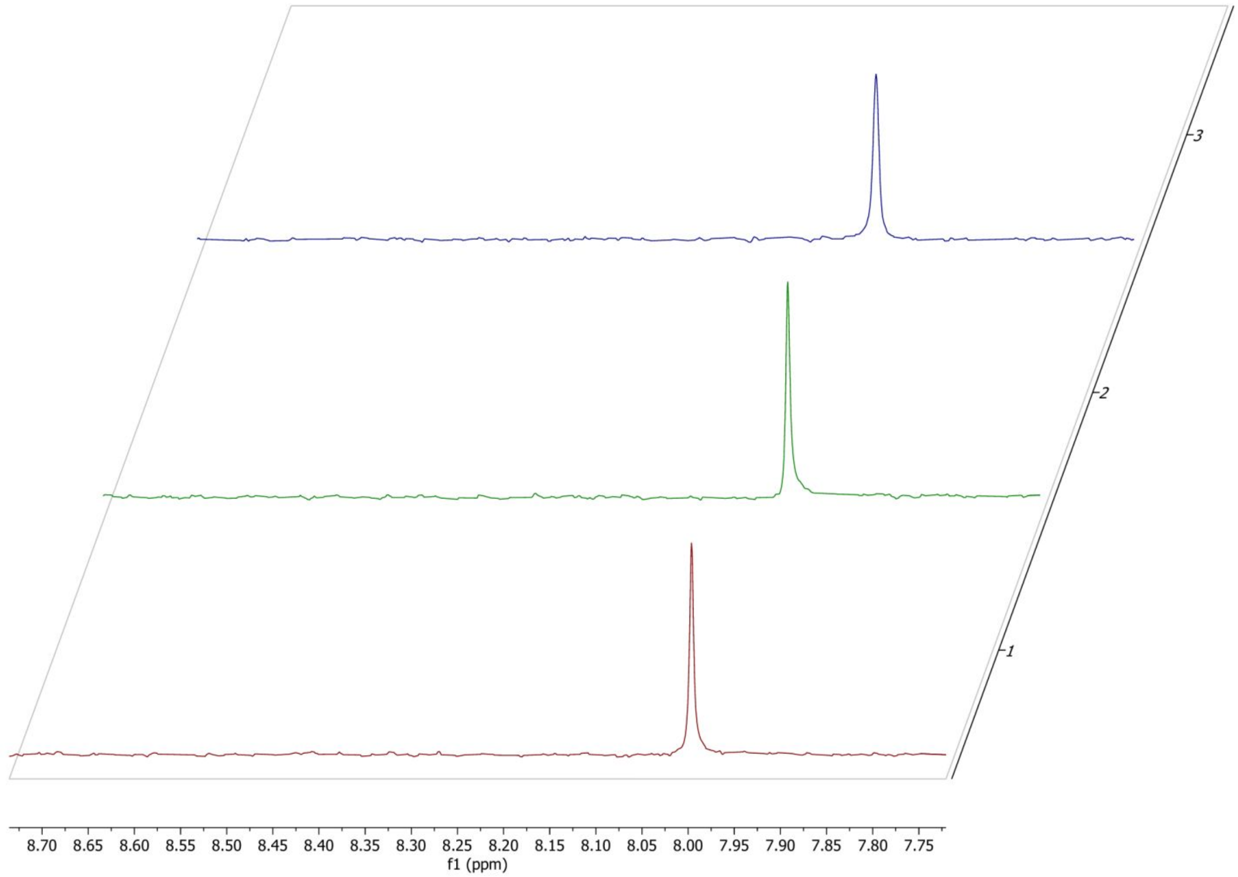

Figure S19: Proton NMR spectra of the reaction of dach-2 with dGMP after 0, 24, and 48 hours (bottom to top). The large peak at 8.0 ppm corresponds to the H8 proton on guanine. The peak at 8.00 ppm corresponds to the H8 proton on guanine, and no additional peaks appear in the aromatic region of the NMR spectrum during this time.

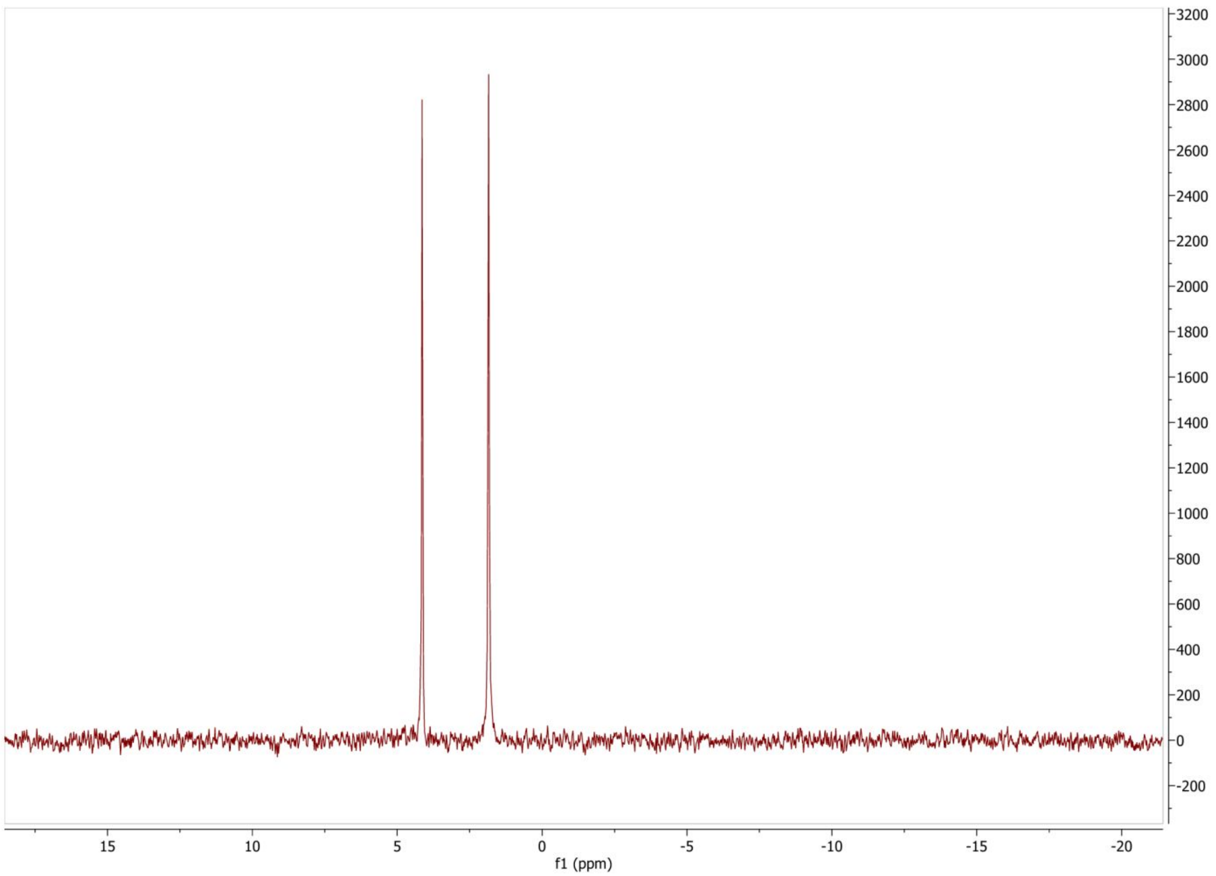

Figure S20: The phosphorus-31 NMR spectrum of the reaction of dach-2 with dGMP following 48 hours shows only the original phosphorus resonances from dach-2 (1.85 ppm) and dGMP (4.14 ppm). No additional resonances are observed.

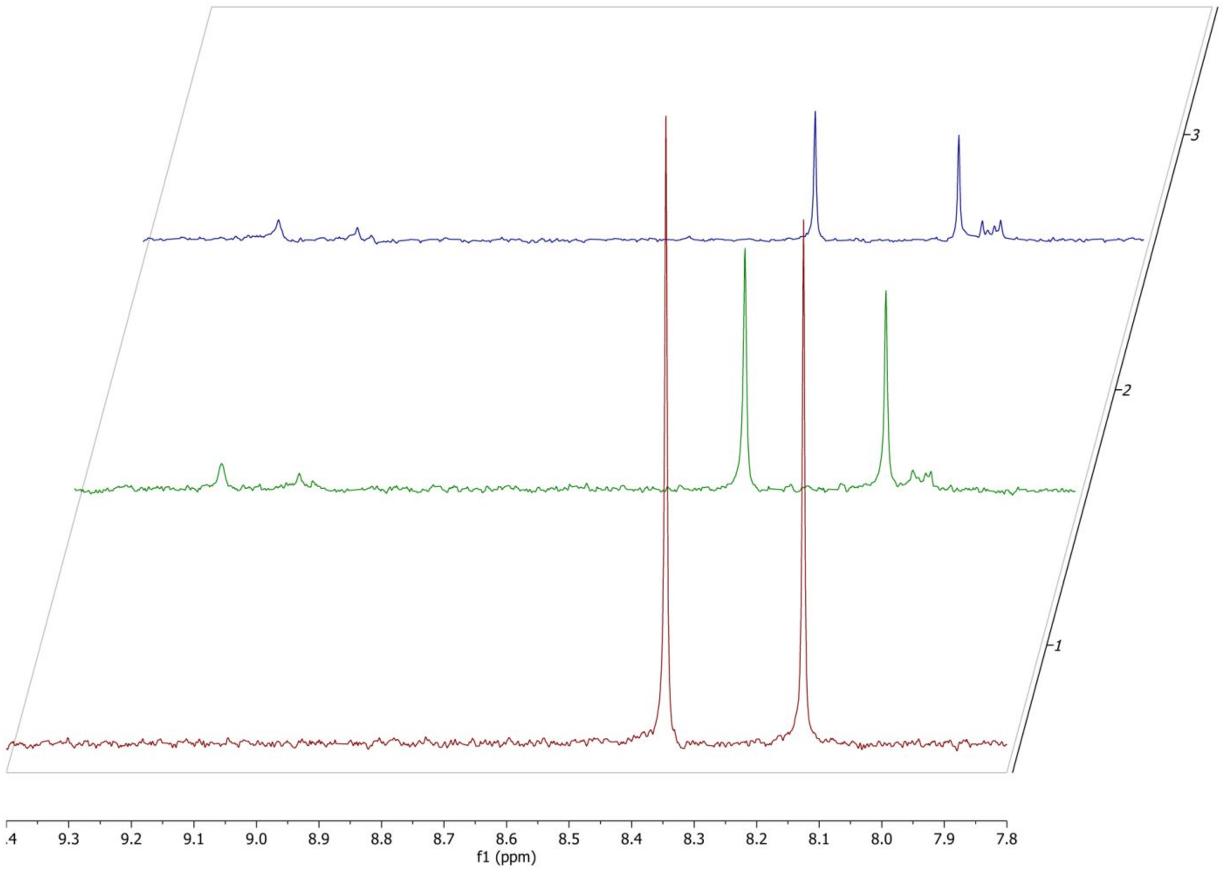

Figure S21: Proton NMR spectra of the reaction of dach-2 with AMP after 0, 24, and 48 hours (bottom to top). Spectral changes were observed within the first 24 hours, indicating the formation of Pt-AMP adducts.

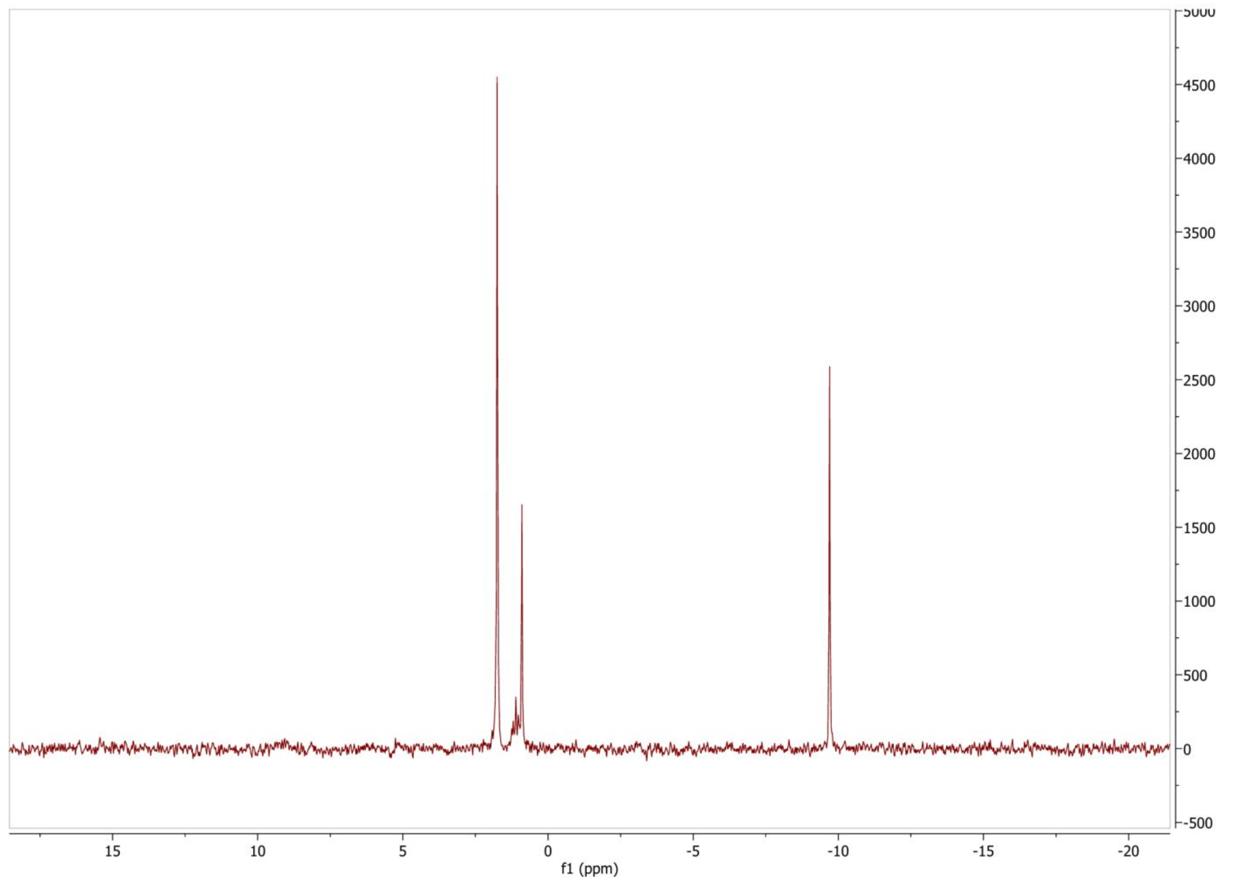

Figure S22: The phosphorus-31 NMR spectrum of the reaction of dach-2 with AMP following 48 shows a new resonance at 1.11 ppm, corresponding to an uncharacterized platinum-bound AMP species. The peaks at 0.90 ppm and 1.75 ppm belong to AMP and dach-2, respectively. A new peak at -9.70 ppm represents pyrophosphate ions that have been liberated from dach-2.

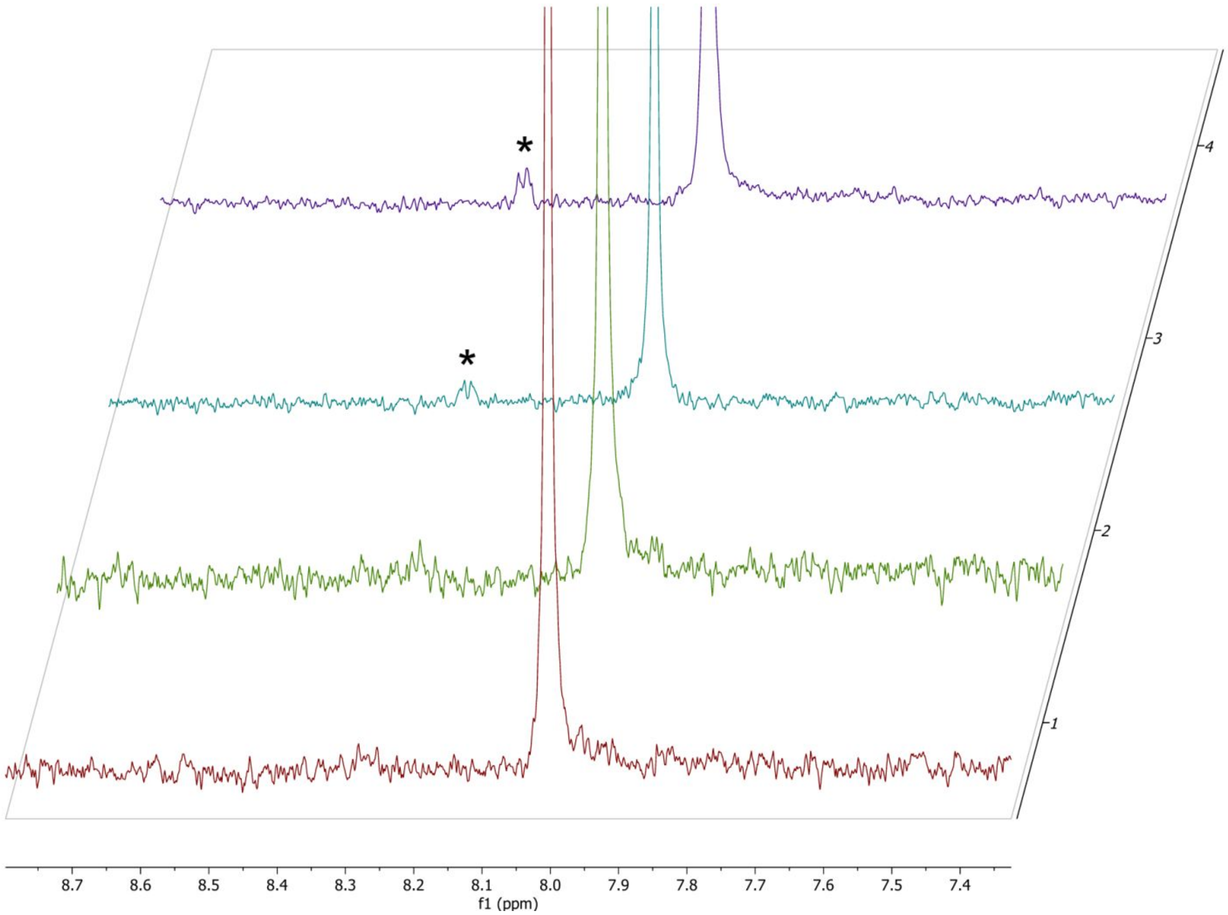

Figure S23: Proton NMR spectra of the reaction of cBuAm-2 with dGMP after 0, 24, 48, and 77 hours (bottom to top). New features can be seen starting at 48 hours and are marked with an asterisk (*).

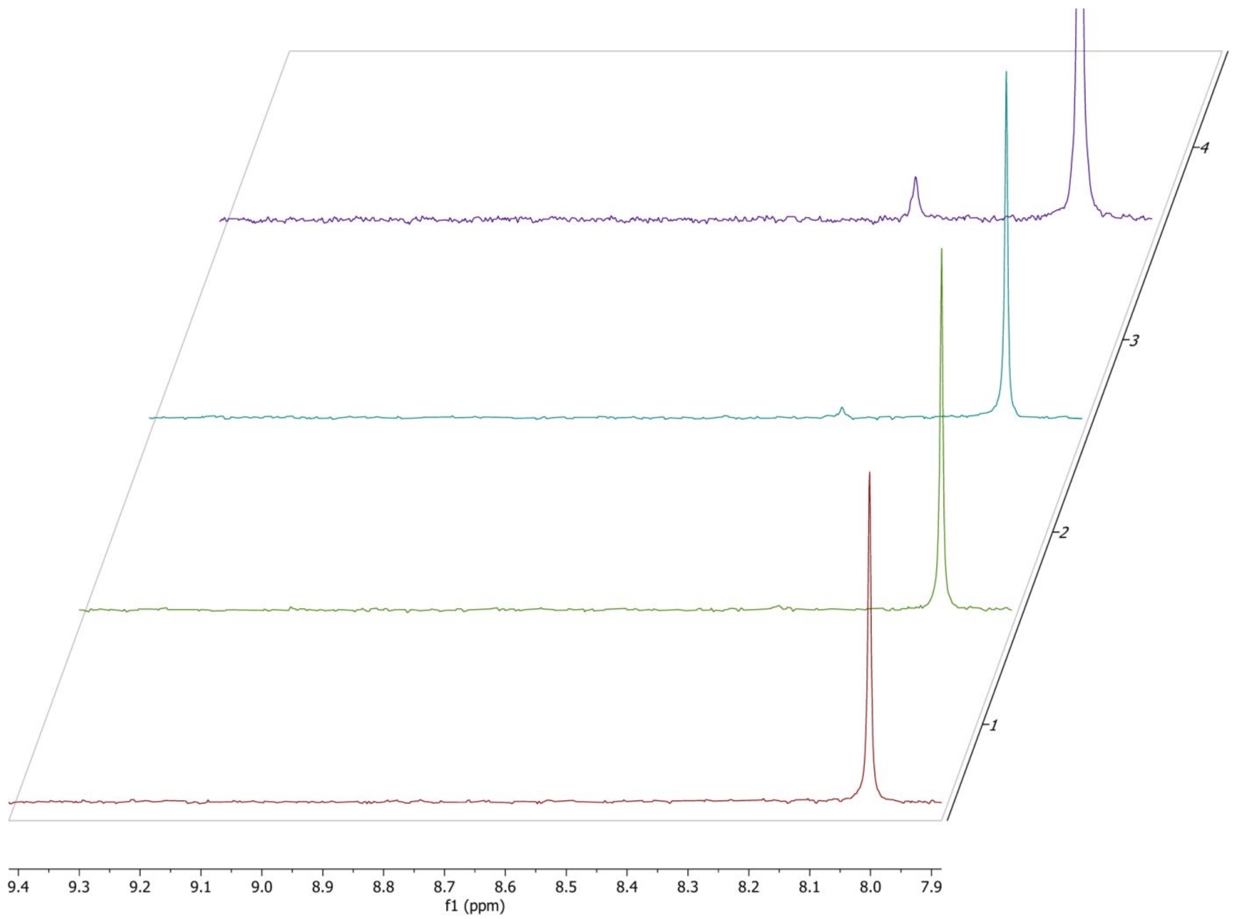

Figure S24: Proton NMR spectra of the reaction of cPnAm-2 with dGMP after 0, 24, 48, and 72 hours (bottom to top). A peak at 8.27 ppm begins to emerge after 24 hours, which grows intensity during the course of the reaction, indicating the formation of Pt-dGMP adducts.

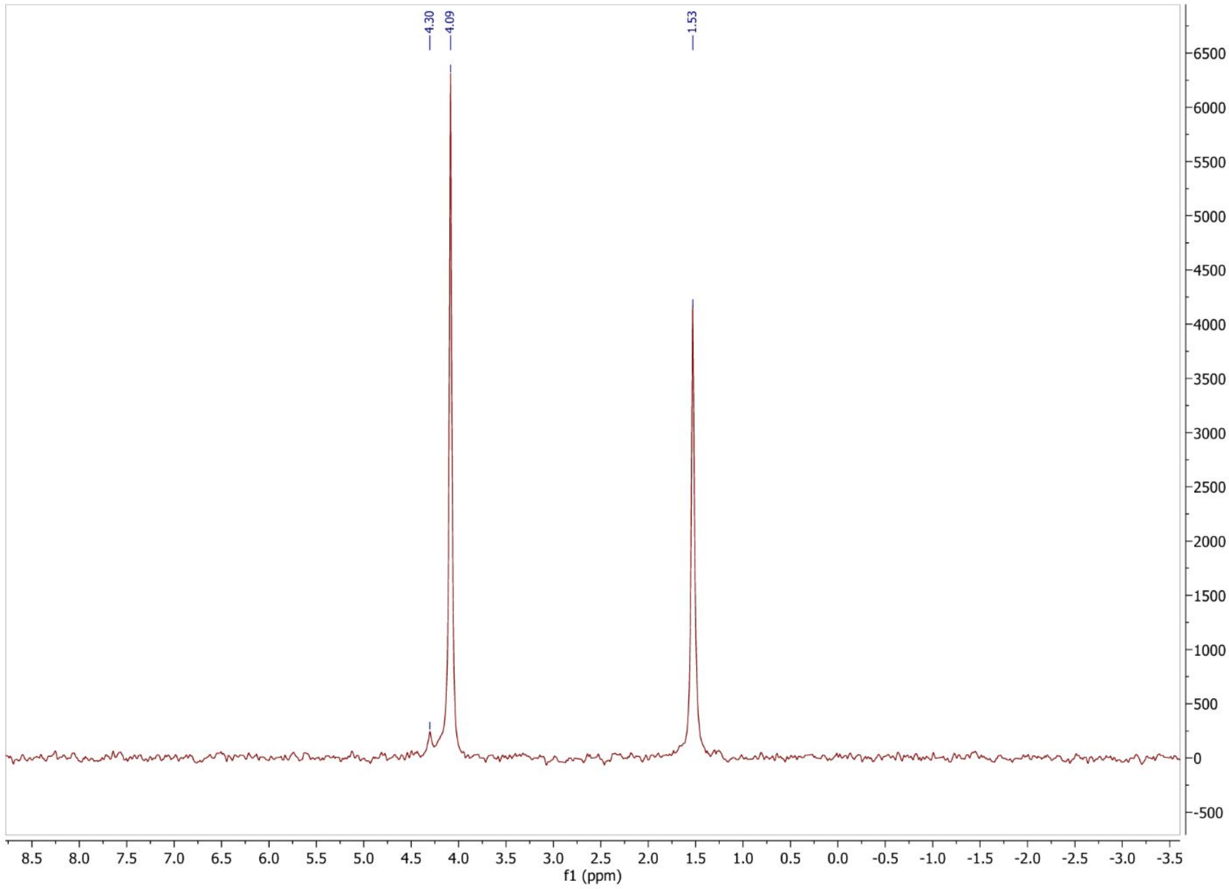

Figure S25: The P-31 NMR spectrum of the reaction of cPnAm-2 with dGMP after 72 hours shows a peak at 4.30 ppm that corresponds to a Pt-dGMP adduct.

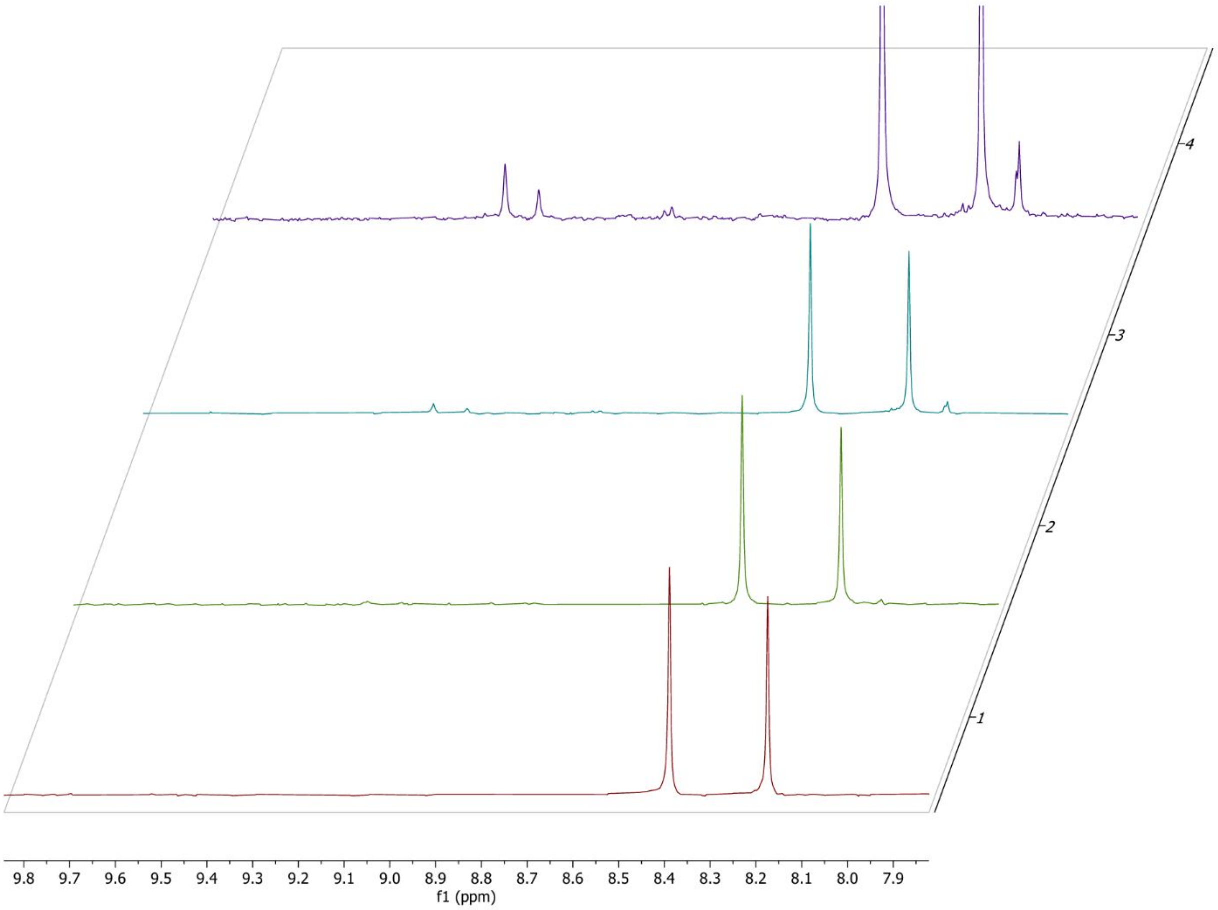

Figure S26: Proton NMR spectra of the reaction of cPnAm-2 with AMP after 0, 24, 48, and 72 hours (bottom to top). New spectral features begin to emerge after 24 hours, which grow intensity during the course of the reaction, indicating the formation of Pt-AMP adducts.

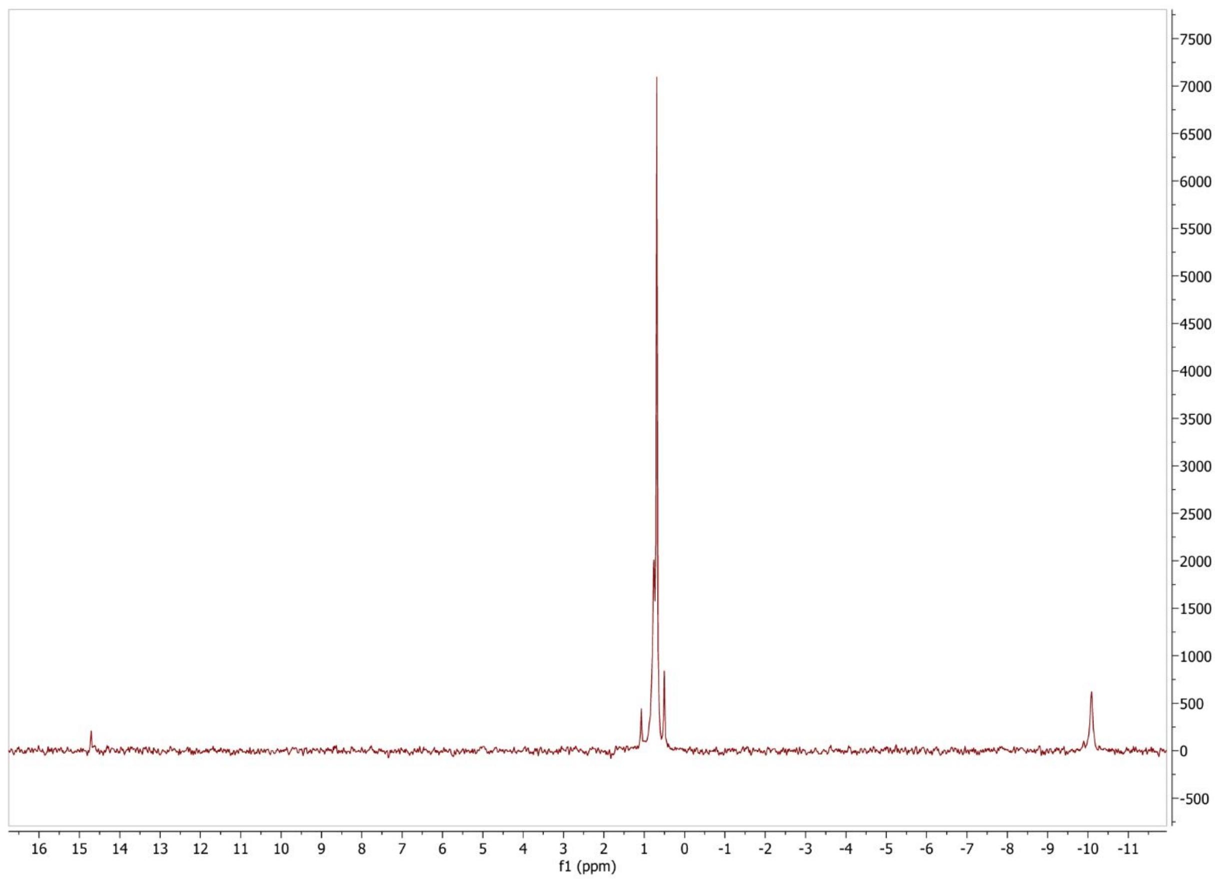

Figure S27: The P-31 NMR spectrum of the reaction of cPnAm-2 with AMP after 72 hours shows new features corresponding to Pt-AMP adducts, as well as peaks that can be assigned as free pyrophosphate (-10 ppm) and a dinuclear platinum species (15 ppm).

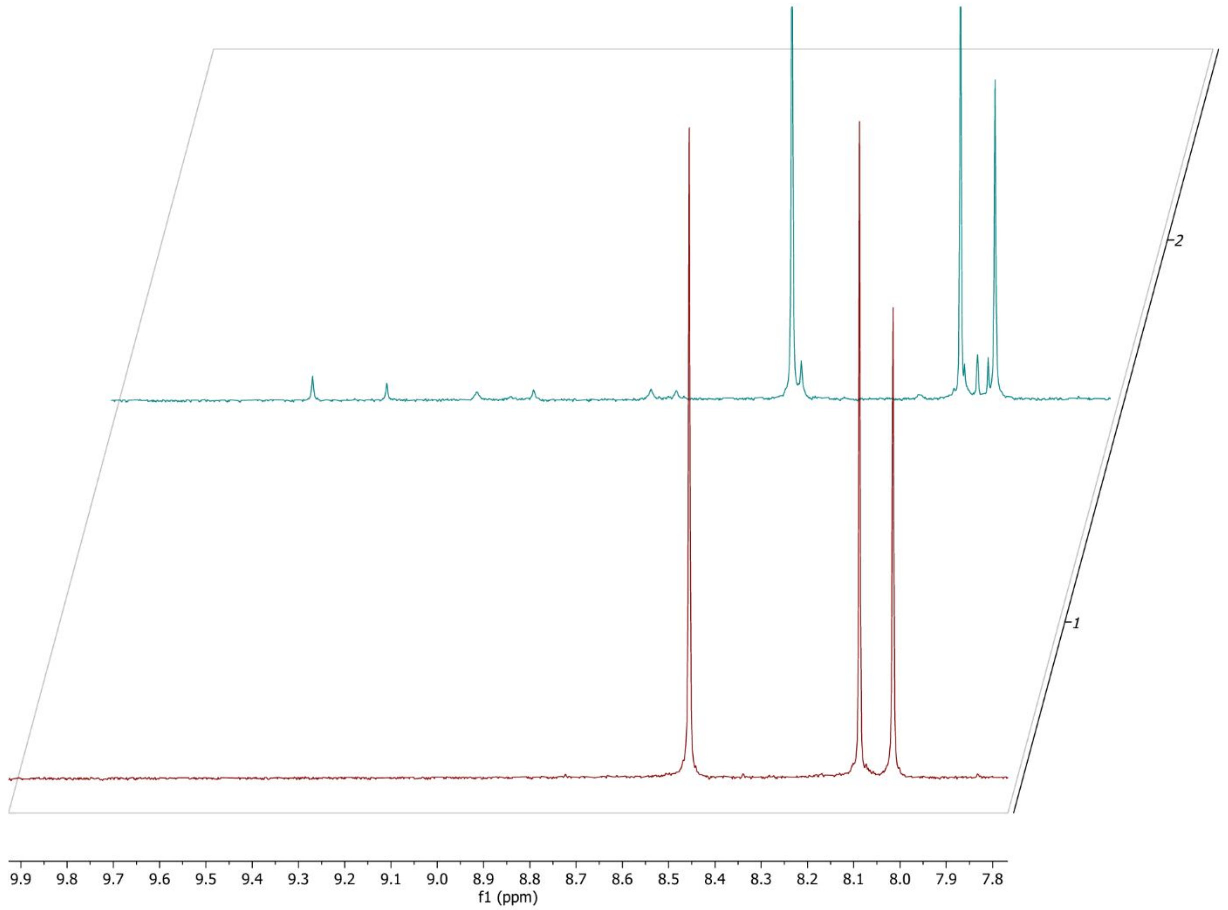

Figure S28: ^{1}H NMR spectra following reaction of cisplatin (5 mM) with a mixture of dGMP and AMP (10 mM each) at 0 hrs (bottom) and 24 hrs (top)

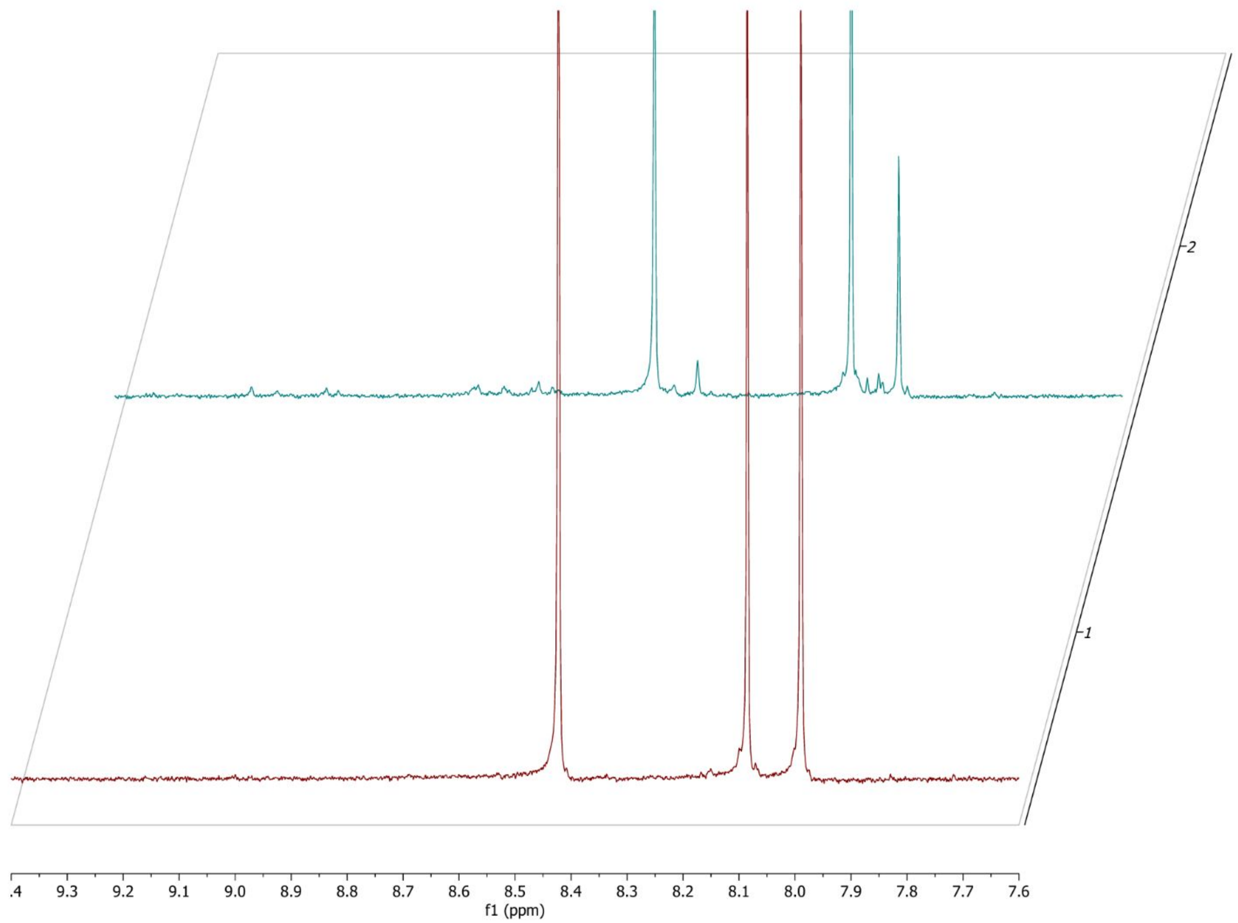

Figure S29: ^{1}H NMR spectra following reaction of dach-2 (5 mM) with a mixture of dGMP and AMP (10 mM each) at 0 days (bottom) and 8 days (top)

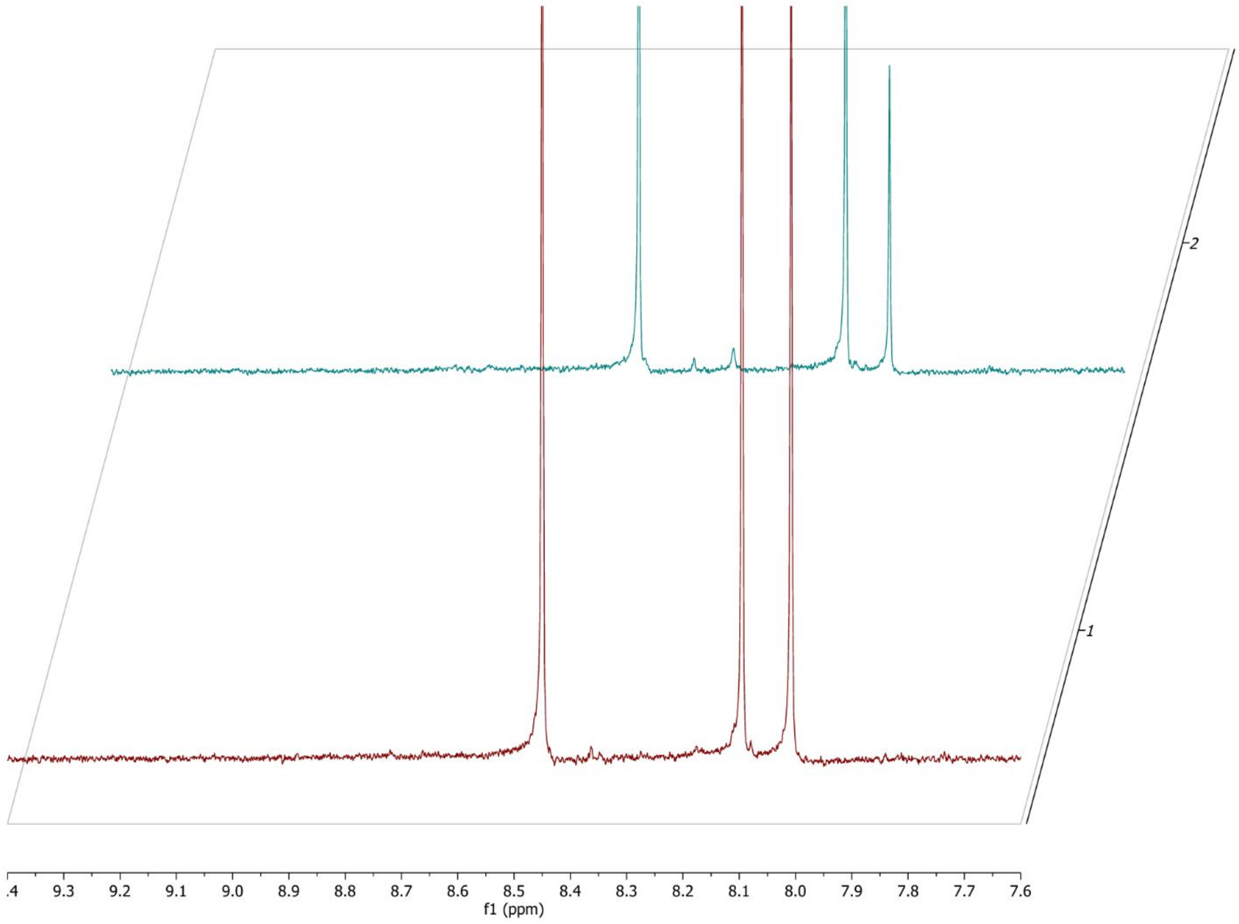

Figure S30: ^{1}H NMR spectra following reaction of cBuAm-2 (5 mM) with a mixture of dGMP and AMP (10 mM each) at 0 days (bottom) and 8 days (top)

Concentration (μM)	Cisplatin	cBuAm-2	cPnAm-2
0	100.0	100	100
3.13	83.0 ± 5.28	N/A	N/A
6.25	72.4 ± 7.33	95.5 ± 9.87	103 ± 5.45
12.5	60.0 ± 7.14	96.0 ± 8.69	105 ± 2.18
25	39.2 ± 6.06	96.9 ± 3.77	104 ± 6.63
50	17.0 ± 5.00	96.5 ± 3.29	91.9 ± 4.73
100	17.3 ± 6.06	78.0 ± 2.06	76.2 ± 2.76
200	N/A	60.9 ± 0.53	61.3 ± 4.95
IC_{50} (μM)	14.4	98.0	85.3

Table S1: Average % viability of MDA-MB-231 cells as determined by MTT cell viability assay. Cells were treated for 48 hours with cisplatin, or 72 hours with either cBuAm-2 or cPnAm-2. Values are reported as a mean % viability of 3 trials ± 1 standard deviation, except for the 200 μM concentration of cBuAm-2, which was the average of two trials. IC_{50} was calculated from the mean % viability for each compound.

Concentration (μM)	Cisplatin	RRD2	cBuAm-2	cPnAm-2
0	100.0	100.0	100.0	100.0
3.13	71.0 ± 6.32	N/A	N/A	N/A
6.25	64.1 ± 3.61	48.9 ± 1.88	91.9 ± 9.36	92.6 ± 8.10
12.5	40.3 ± 6.38	46.7 ± 1.40	89.9 ± 0.95	79.9 ± 12.4
25.0	20.0 ± 2.55	45.5 ± 1.54	79.8 ± 5.30	61.0 ± 2.05
50.0	19.2 ± 4.71	42.3 ± 2.14	55.6 ± 11.3	57.8 ± 6.72
100	18.4 ± 3.82	31.1 ± 4.21	45.9 ± 6.05	48.3 ± 6.86
200	N/A	13.6 ± 2.40	32.5 ± 4.84	31.2 ± 4.35
IC_{50} (μM)	6.81	1.34	44.6	36.3

Table S2: Average % viability of A549 cells as determined by MTT cell viability assay. Cells were treated for 48 hours with cisplatin, or 72 hours with cBuAm-2, cPnAm-2, or RRD2. Values are reported as a mean % viability of 3 trials ± 1 standard deviation. IC_{50} was calculated from the mean % viability for each compound.

Concentration (μM)	RRD2
0	100.0
0.27	96.0 ± 0.44
0.82	89.7 ± 3.48
2.47	76.6 ± 172
7.41	62.0 ± 1.92
22.2	53.9 ± 2.92
66.7	52.5 ± 2.60
200	45.9 ± 2.50
IC_{50} (μM)	3.07

Table S3: Average % viability of MDA-MB-231 cells as determined by MTT cell viability assay. Cells were treated for 72 hours with RRD2. Values are reported as a mean % viability of 3 trials ± 1 standard deviation. IC_{50} was calculated from the mean % viability for each compound.

Concentration (μM)	RRD2
0	100.0
0.27	96.8 ± 3.38
0.82	74.8 ± 3.76
2.47	45.1 ± 1.89
7.41	39.3 ± 1.75
22.2	37.3 ± 1.49
66.7	31.9 ± 1.99
200	11.7 ± 1.05
IC_{50} (μM)	1.34

Table S4: Average % viability of A549 cells as determined by MTT cell viability assay. Cells were treated for 72 hours with RRD2. Values are reported as a mean % viability of 3 trials ± 1 standard deviation. IC_{50} was calculated from the mean % viability for each compound.
